# Lenvatinib or anti-VEGF in combination with anti–PD-1 differentially augments antitumor activity in melanoma

**DOI:** 10.1172/jci.insight.157347

**Published:** 2023-04-10

**Authors:** Thuy T. Tran, Jasmine Caulfield, Lin Zhang, David Schoenfeld, Dijana Djureinovic, Veronica L. Chiang, Victor Oria, Sarah A. Weiss, Kelly Olino, Lucia B. Jilaveanu, Harriet M. Kluger

**Affiliations:** 1 Yale School of Medicine and Yale Cancer Center, New Haven, Connecticut, USA.; 2 Yale School of Medicine, Department of Neurosurgery, New Haven, Connecticut, USA.; 3 Yale School of Medicine, Department of Surgery, New Haven, Connecticut, USA.

**Keywords:** Oncology, Cancer, Cancer immunotherapy, Melanoma

## Abstract

Targeting tumor-associated blood vessels to increase immune infiltration may enhance treatment effectiveness, yet limited data exist regarding anti-angiogenesis effects on the tumor microenvironment (TME). We hypothesized that dual targeting of angiogenesis with immune checkpoints would improve both intracranial and extracranial disease. We used subcutaneous and left ventricle melanoma models to evaluate anti–PD-1/anti-VEGF and anti–PD-1/lenvatinib (pan-VEGFR inhibitor) combinations. Cytokine/chemokine profiling and flow cytometry were performed to assess signaling and immune-infiltrating populations. An in vitro blood-brain barrier (BBB) model was utilized to study intracranial treatment effects on endothelial integrity and leukocyte transmigration. Anti–PD-1 with either anti-VEGF or lenvatinib improved survival and decreased tumor growth in systemic melanoma murine models; treatment increased Th1 cytokine/chemokine signaling. Lenvatinib decreased tumor-associated macrophages but increased plasmacytoid DCs early in treatment; this effect was not evident with anti-VEGF. Both lenvatinib and anti-VEGF resulted in decreased intratumoral blood vessels. Although anti-VEGF promoted endothelial stabilization in an in vitro BBB model, while lenvatinib did not, both regimens enabled leukocyte transmigration. The combined targeting of PD-1 and VEGF or its receptors promotes enhanced melanoma antitumor activity, yet their effects on the TME are quite different. These studies provide insights into dual anti–PD-1 and anti-angiogenesis combinations.

## Introduction

Melanoma has the highest predisposition to form brain metastases (BrMs) in advanced disease compared with all other malignancies (1). Immune checkpoint inhibitors (ICIs) have emerged as the central treatment for advanced melanoma, and only within the last 5 years have phase II clinical trials demonstrated intracranial efficacy of these agents (2). The most effective ICI regimen combining ipilimumab and nivolumab has response rates approaching 58%, but 59% of individuals develop grade 3–4 serious adverse events (3). For those who fail to respond initially, progress on treatment, or develop treatment-limiting toxicities such as edema or radiation necrosis, alternative approaches are urgently needed. Those with active, previously untreated BrMs have further therapeutic limitations, as many trials only permit those with small, asymptomatic metastases or previously irradiated lesions. There is an immediate and unmet need for alternative strategies that penetrate the blood-brain barrier (BBB) and improve ICI activity without worsening toxicity.

Recent evidence suggests a synergistic role for dual ICI and angiogenesis targeting (4, 5). Tumors exploit enhanced VEGF signaling to evade the immune system and to facilitate growth by increasing angiogenesis (6). High circulating VEGF levels are associated with decreased survival in patients with melanoma treated with either high-dose IL-2 or ipilimumab, thus, suggesting that soluble VEGF can serve as a biomarker of clinical outcome and a potential target to reverse the immunosuppressive tumor microenvironment (TME) (7, 8). VEGF promotes Treg and myeloid-derived suppressor cell (MDSC) expansion, inhibits DC maturation, mitigates effector T cell responses, and alters lymphocyte trafficking into tumors (9–14). Conversely, angiogenesis inhibitors promote T cell infiltration and reduce immune-suppressive tumor-associated macrophages (TAMs) (15, 16). In extracerebral melanoma metastases, ipilimumab and bevacizumab (anti-VEGF) increased CD8^+^ T cell and macrophage infiltration (7). Bevacizumab is a humanized monoclonal Ab, but because of its prolonged half-life, side effects such as gastrointestinal perforation, hemorrhage, and blood clots may be difficult to treat and/or require permanent discontinuation. Bevacizumab is specific for VEGF-A and does not crossreact with other VEGF isoforms, thus having a more specific role in mitigating VEGFR-2 signaling. Furthermore, resistance via autocrine and intracrine VEGF and VEGFR-2 signaling can develop with bevacizumab (17).

An alternative approach to VEGF inhibition (VEGFi) is VEGFRi with lenvatinib. Lenvatinib is a multiple tyrosine kinase inhibitor of VEGFR1–3, FGF receptors 1–4 (FGFR1–4), PDGFRα, KIT, and RET (18). In vitro kinase inhibitory activity is highest against VEGFR2 followed by VEGFR3, VEGFR1, RET, KIT, and then FGFRs (19). It is unclear which receptors are important in mediating lenvatinib’s antitumor effect. Signaling through VEGFRs and FGFRs promotes tumor-associated angiogenesis (20, 21), and FGFR activation results in decreased Tregs, programmed death ligand-1 (PD-L1) expression, and increased IFN-γ^+^CD8^+^ T cells (22, 23). Lenvatinib can be dose adjusted for tolerability given its shorter half-life and is known to modify the immune milieu by decreasing TAM populations (24). The combination of pembrolizumab and lenvatinib is FDA approved in the second-line treatment of advanced endometrial carcinomas (5) and for advanced renal cell carcinomas (4) and is being tested in the front- and second-line settings for melanoma (LEAP-003 [NCT03820986] and LEAP-004 [NCT03776136]), where it has demonstrated early efficacy (25). Little is known about effects of inhibiting VEGF versus its receptor on altering immune infiltration and endothelial cells, particularly when combined with concurrent ICI therapy. Additionally, bevacizumab has been shown to limit brain radiation necrosis and edema, but little is known about how lenvatinib affects these toxicities (26, 27). Even less is known regarding the efficacy of these agents in BrMs and their impact specifically on the brain TME.

We assessed the efficacy of anti–PD-1 combined with either anti-VEGF or lenvatinib in immune-competent murine models of local and disseminated melanoma and profiled tumor-infiltrating leukocytes and circulating cytokines/chemokines. Because melanoma is highly cerebrotropic, the effect of these drug combinations on leakiness in an in vitro BBB model and on transendothelial migration of PBMCs was also assessed. We hypothesized that both anti-VEGF and anti-VEGFR combined with anti–PD-1 would result in a more robust immune response than anti–PD-1 alone. These studies demonstrate the overall effectiveness of concurrent anti–PD-1 and angiogenesis targeting and highlight alternative immune pathways utilized in mediating the antitumor effect of anti-VEGF versus anti-VEGFR. Our results provide preclinical rationale for the continued development of these combinations.

## Results

### Lenvatinib directly inhibits cell viability in melanoma cultures.

Given the potential of lenvatinib to inhibit cell viability, short-term melanoma cultures were exposed to increasing concentrations of lenvatinib or bevacizumab. Lenvatinib demonstrated in vitro melanoma cytotoxicity in 72% (13 of 18) of short-term cultures at a mean concentration of 10.7 ± 6.4 μM (range 3.3–28.5 μM), whereas no cytotoxicity was observed with doses up to 300 μg/mL of bevacizumab (Supplemental Table 1; supplemental material available online with this article; https://doi.org/10.1172/jci.insight.157347DS1).

**Figure 1:**
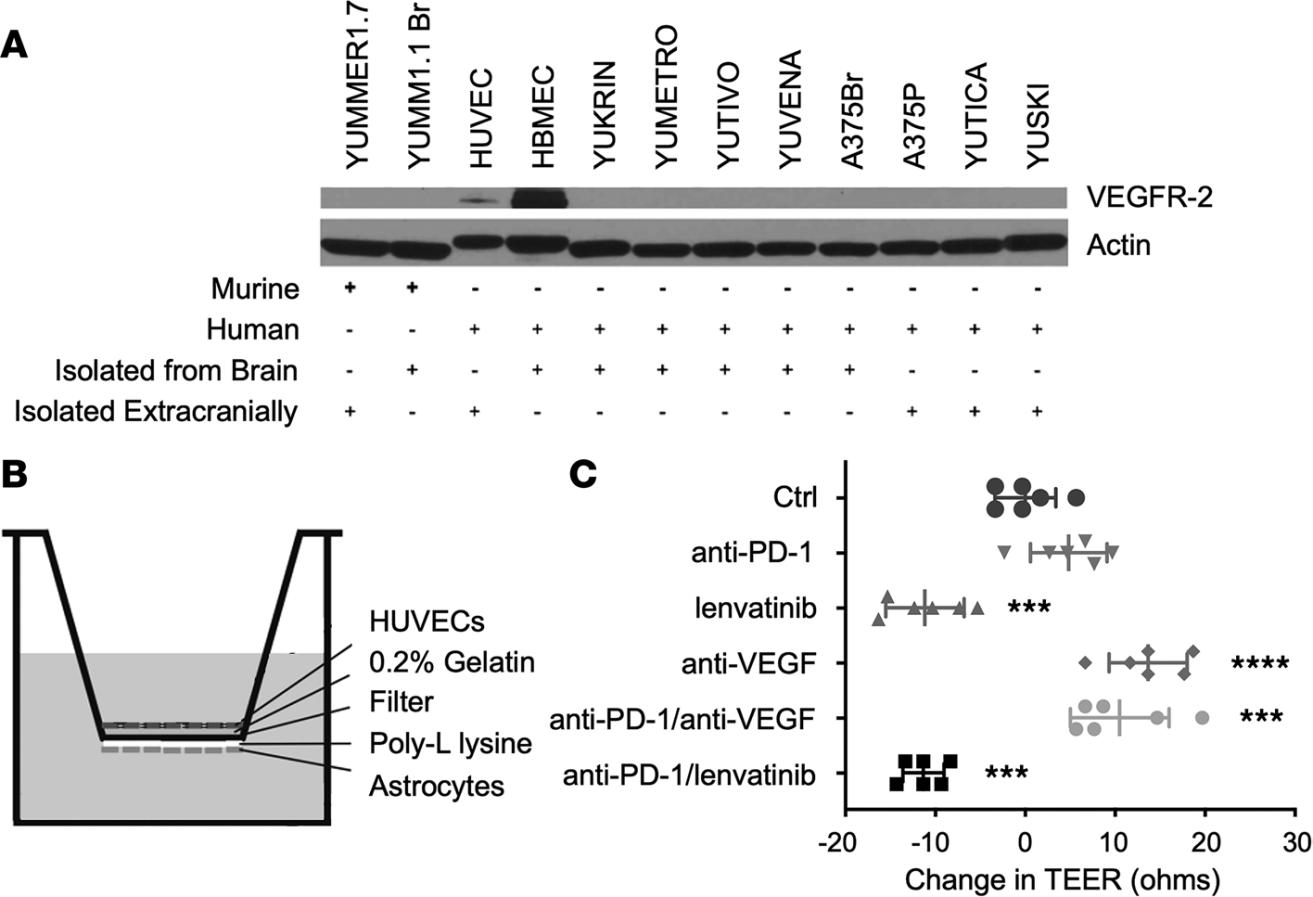
Lenvatinib induces increased brain vascular permeability compared with bevacizumab. (**A**) VEGFR-2 is not expressed on murine or short-term cultures of human melanoma cells. VEGFR-2 is highly expressed on HBMECs at increased levels compared with extracranial HUVECs, thus, suggesting increased potential sensitivity of brain endothelium to VEGFR-2 targeting agents. (**B**) Diagram of the in vitro BBB setup. (**C**) TEER measures endothelial tight junction integrity and is reduced with lenvatinib treatment but increased with anti-VEGF treatment in the in vitro BBB model. Treatment with anti–PD-1 did not impact TEER. ***P < 0.001 and ****P < 0.0001, 1-way ANOVA with correction for multiple comparisons.

Although VEGFR-2 is the main endothelial cell receptor associated with angiogenesis, some prior studies suggest melanoma cells can express VEGFR-2 (28). We found strong VEGFR-2 expression in human brain microvasculature endothelial cells (HBMEC) compared with HUVECs, whereas melanoma cells did not express any detectable levels (Figure 1A). This suggests that the mechanism for direct lenvatinib cytotoxicity of melanoma cells is VEGFR-2 independent and likely results from inhibition of other lenvatinib targets.

### Lenvatinib increased brain endothelial permeability, whereas anti-VEGF did not.

To compare the effect of lenvatinib and bevacizumab on the endothelial cells of the brain, we modified a well-established in vitro BBB transwell assay to evaluate drug effects on endothelial barrier function (Figure 1B). Although HUVECs are extracranial endothelial cells, their ease of use, reproducibility, and ability to acquire brain endothelial cell characteristics and tight junctions make them a superior source of endothelial cells for higher throughput BBB screening (29, 30). We measured transendothelial electrical resistance (TEER) as an established surrogate for tight junction leakiness. The addition of anti–PD-1 onto the in vitro BBB did not result in any change in TEER after 24 hours compared with untreated control. In the presence of lenvatinib, TEER decreased, indicating defects in endothelial tight junctions. However, in the presence of anti-VEGF, TEER increased, indicating enhanced endothelial barrier function. The TEER changes seen with lenvatinib or anti-VEGF were not impacted by the addition of anti–PD-1 (Figure 1C). Of note, we assessed concentrations of lenvatinib ranging from 1–10 μM (data not shown), and the results in Figure 1C were obtained with the 1 μM dose, as it was closest to patient plasma concentrations (31) and limited direct cell cytotoxicity (Supplemental Table 2).

### Combined anti-angiogenic therapies and anti–PD-1 enhanced antitumor responses, survival, and memory responses in an immune-sensitive extracranial melanoma model.

**Figure 2:**
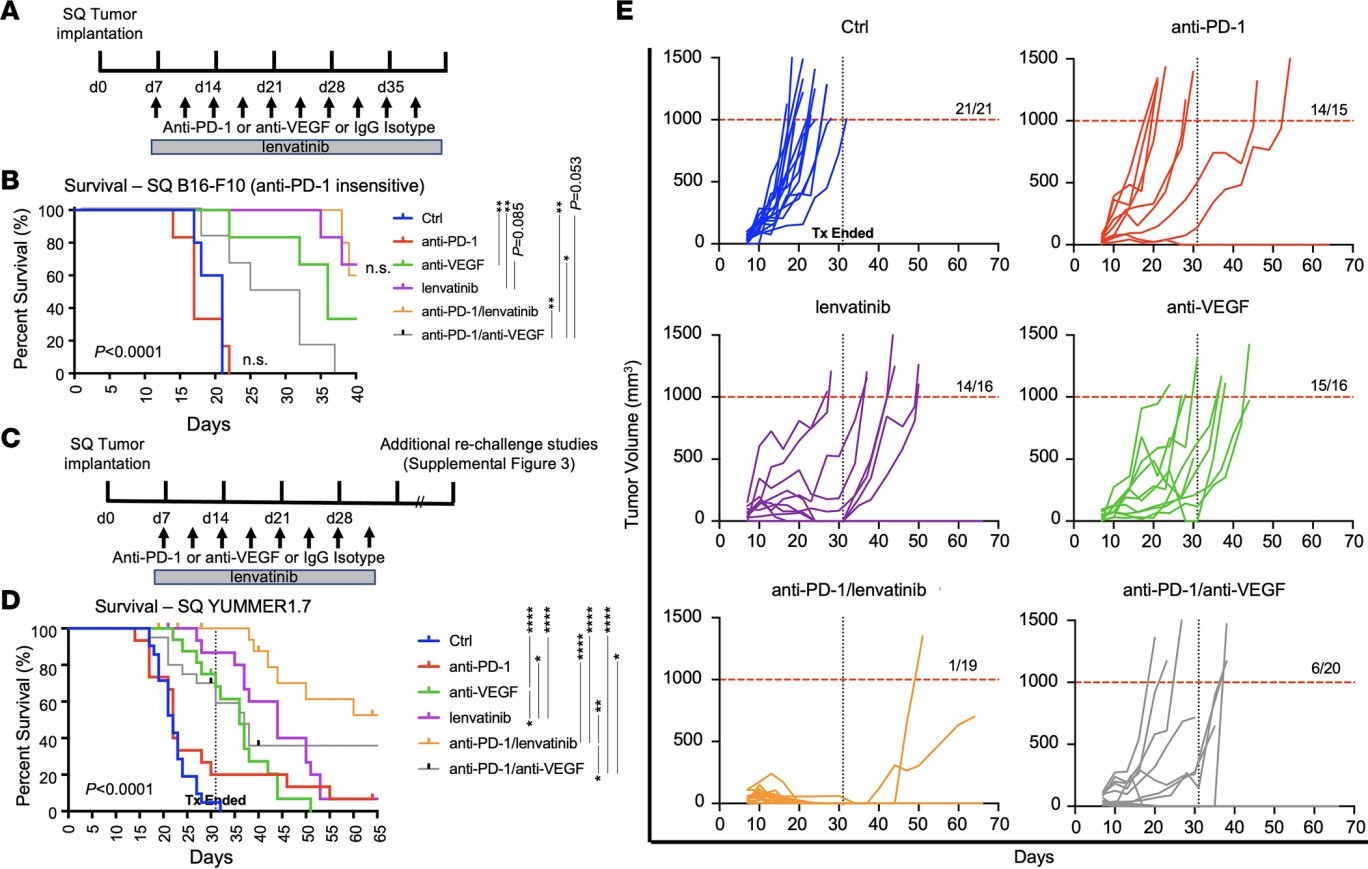
Combined antivascular directed therapies and anti–PD-1 enhance antitumor responses, duration of response, and memory responses in extracranial animal models of melanoma. (**A**) Experimental timeline for B16-F10 melanoma s.c. (SQ) experiments. (**B**) Mice with B16-F10 s.c. tumors were followed for 40 days after tumor implantation (*n* = 6 in all treatment groups except for control and anti–PD-1/lenvatinib, where *n* = 5). Anti–PD-1 had no benefit compared with control or when combined with lenvatinib or anti-VEGF, demonstrating that B16-F10 is an anti-PD-1–insensitive melanoma model that does not recapitulate human disease well. (**C**) Experimental timeline for YUMMER1.7 melanoma SQ experiments. Last dose of treatment was given on day 31, and animals were followed for tumor regrowth. Animals that remained tumor free were rechallenged to test for memory responses (Supplemental Figure 3). (**D**) Survival curve of SQ YUMMER1.7 animals demonstrates prolonged survival of anti–PD-1/lenvatinib-treated mice, which was superior to anti–PD-1/anti-VEGF–treated animals. Monotherapy with either anti–PD-1, anti-VEGF, or lenvatinib only resulted in modest improvements in survival compared with isotype control. (**E**) Individual tumor volume measurements for animals implanted with YUMMER1.7 cells. Endpoint was defined as a tumor of greater than 1,000 mm^3^. *P < 0.05, **P < 0.01, and ****P < 0.0001, log-rank testing.

We employed B16-F10 and YUMMER1.7 melanoma models to study the effect of lenvatinib and anti-VEGF in immune-competent murine models. Viability of both cell types was inhibited by lenvatinib, with an IC_50_ of 5.5 μM in B16-F10 and 3.5 μM in YUMMER1.7 cells. Neither cell line was directly inhibited by anti-VEGF (range 0–300 μg/mL) (Supplemental Table 3). C57Bl6 mice were s.c. injected with 3 x 10^5^ B16-F10 cells and treated twice a week with a monoclonal Ab and/or daily with lenvatinib starting on day 7 after injection (Figure 2A). In the immune-insensitive B16-F10 melanoma model, treatment with anti–PD-1 did not result in an improvement in survival, defined as the time until s.c.-implanted tumors reached 1,000 mm^3^. The addition of anti–PD-1 to lenvatinib did not improve survival compared with lenvatinib alone (*P =* 0.98). The combination of anti–PD-1/anti-VEGF resulted in slightly inferior survival compared with anti-VEGF alone (*P* = 0.043). Anti–PD-1 with lenvatinib was superior to anti–PD-1/anti-VEGF (*P* = 0.0015) (Figure 2B and Supplemental Figure 1).

One of the limitations of the B16-F10 model is a lack of genetic alternations seen in human melanoma. We therefore employed the YUMMER1.7 melanoma cell line, which has a mutation in *BRAF* and loss of *PTEN* and *CDKN2A* along with additional radiation-induced mutations. This model is more responsive to ICIs. Mice were s.c.-injected with 3 x 10^5^ YUMMER1.7 cells and treated twice a week with a monoclonal Ab and/or daily lenvatinib starting on day 7 until day 31, as responding tumors generally had fully regressed by this time point (Figure 2C). Anti–PD-1 monotherapy produced a slight benefit in survival compared with control, which was not statistically significant (*P* = 0.18). The addition of anti–PD-1 to anti-VEGF further improved survival, but the combination of anti–PD-1 and lenvatinib resulted in the best survival outcomes (anti–PD-1/lenvatinib versus anti–PD-1/anti-VEGF, *P* = 0.013; *P* < 0.0001 for control versus either anti–PD-1/lenvatinib or anti–PD-1/anti-VEGF) (Figure 2D). Tumor growth curves are shown in Figure 2E, demonstrating superiority of anti–PD-1/lenvatinib to other treatment groups, followed by the combination of anti–PD-1/anti-VEGF, with 1/19 and 6/20 mice requiring sacrifice for tumors larger than or equal to 1,000 mm^3^. Interestingly, lenvatinib monotherapy resulted in a quick reduction of tumor volume by day 31 but many nonpalpable lesions regrew after withdrawal of treatment. The addition of anti–PD-1 to lenvatinib helped promote ongoing antitumor responses even after withdrawal of treatment (Figure 2E). To compare tumor growth curves, the AUC by day 17 was assessed and demonstrated significant tumor growth reduction when anti–PD-1 was combined with either anti-VEGF or lenvatinib, *P* < 0.001 and < 0.0001, respectively, compared with control (Supplemental Figure 2).

To test for antitumor memory responses, we rechallenged animals beyond day 70 with 5 x 10^5^ YUMMER1.7 melanoma cells (Supplemental Figure 3A). Animals were not retreated. All rechallenged animals demonstrated complete rejection of tumor cells by day 15 following injection (Supplemental Figure 3B). All animals were then rechallenged with 1 x 10^5^ YUMMER1.7 cells injected into the left ventricle (LV) to simulate metastatic disease dissemination, including intracranial metastases. Intracranial and extracranial disease burden was assessed via in vivo imaging system (IVIS) bioluminescence but showed no change in luminescence signal to suggest tumor growth (Supplemental Figure 3C).

### Combined anti-angiogenic therapies and anti–PD-1 enhanced antitumor responses, survival, and memory responses in a model of metastatic melanoma.

**Figure 3:**
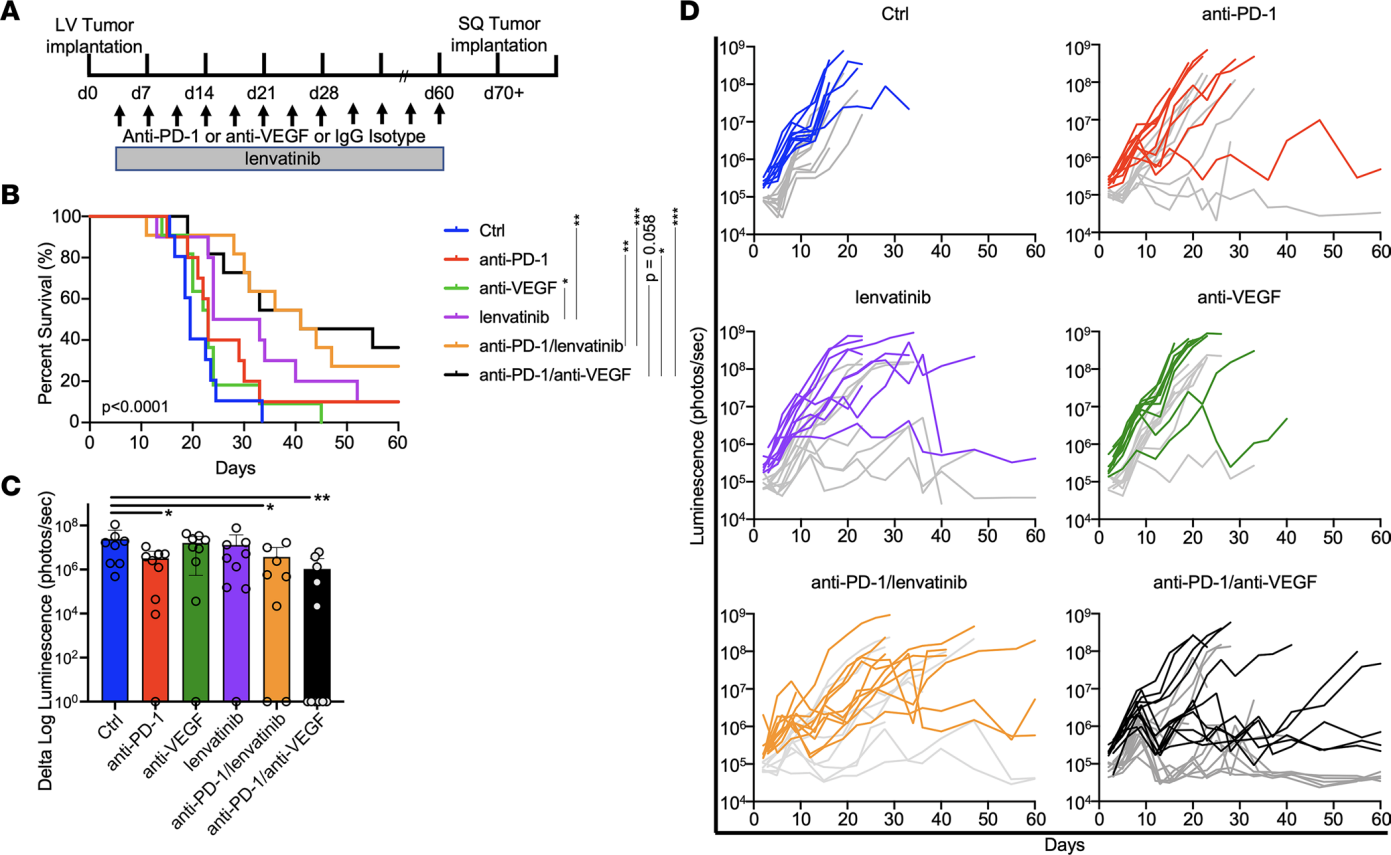
Anti–PD-1 combined with lenvatinib or anti-VEGF enhanced intracranial antitumor responses in a metastatic animal model of melanoma. (**A**) Experimental timeline for YUMMER1.7 melanoma injected into the LV to model metastatic disease, including BrMs. Mice were injected on day 0 and treatment initiated on day 3. (**B**) Survival was lower in the LV model due to increased aggressiveness of metastatic disease compared with the SQ model. Treatment with lenvatinib alone and especially with anti–PD-1/lenvatinib or anti–PD-1/anti-VEGF resulted in improved survival of animals compared with control. There was no significant difference between the groups receiving anti–PD-1/lenvatinib or anti–PD-1/anti-VEGF. As monotherapy, lenvatinib was superior to anti-VEGF. Curves compared by log-rank testing. (**C**) The decrease in intracranial luminescence was significant between control and anti–PD-1 alone, anti–PD-1/lenvatinib, and anti–PD-1/anti-VEGF at day 16 by 2-way ANOVA with correction for multiple comparisons. (**D**) Individual animal luminescence in each treatment arm graphed over time. Dark colored lines represent whole body ventral luminescence. Gray lines represent dorsal cranial luminescence. *n* = 9 in the control, anti-VEGF, and anti–PD-1/lenvatinib cohorts. *n* = 10 in the anti–PD-1 and lenvatinib cohorts. *n* = 11 in the anti–PD-1/anti-VEGF cohorts. *P < 0.05, **P < 0.01, and ***P < 0.001.

To evaluate the efficacy of these drug combinations in disseminated disease, 1 x 10^5^ YUMMER1.7 cells were directly injected into the LV of C57Bl6 mice. Given the increased aggressiveness of the LV model, animals were treated starting on day 3 following injection and luminescence imaged twice a week (Figure 3A; and Supplemental Figure 4, A and B). LV injections of YUMMER1.7 resulted in widespread dissemination of tumor to all major organs, including the brain (Supplemental Figure 4, C and D). Treatment with single-agent anti–PD-1 or anti-VEGF did not prolong survival compared with control animals, whereas lenvatinib did (*P* = 0.0046). While effective at reducing the intracranial IVIS signal, anti–PD-1 therapy did not impact survival, likely owing to progression of extracranial disease that drove survival outcomes in this model. Survival was significantly improved in animals that received dual anti–PD-1 and anti-angiogenic therapies (*P* < 0.001 for control versus either anti–PD-1/lenvatinib or anti–PD-1/anti-VEGF; Figure 3B). Both anti–PD-1/lenvatinib and anti–PD-1/anti-VEGF were effective without a significant difference between the 2 groups (*P* = 0.70). Measurement of intracranial luminescence demonstrates a reduction in signal with anti–PD-1, anti–PD-1/lenvatinib, and anti–PD-1/anti-VEGF treatment compared with control by day 16 (*P* = 0.023, 0.031, and 0.0095, respectively) (Figure 3, C and D). Extracranial luminescence also decreased similar to intracranial signal, with a strong Pearson’s correlation (*r* = 0.88, *P* < 0.0001) between intracranial and whole body luminescence (Supplemental Figure 4, E and F). Animals alive by day 70 were rechallenged in the flank with 5 x 10^5^ YUMMER1.7 melanoma cells, and all demonstrated complete rejection of tumor in the absence of re-treatment.

### Anti–PD-1 with either lenvatinib or anti-VEGF therapies work by enhancing Th1 cytokine responses.

**Figure 4:**
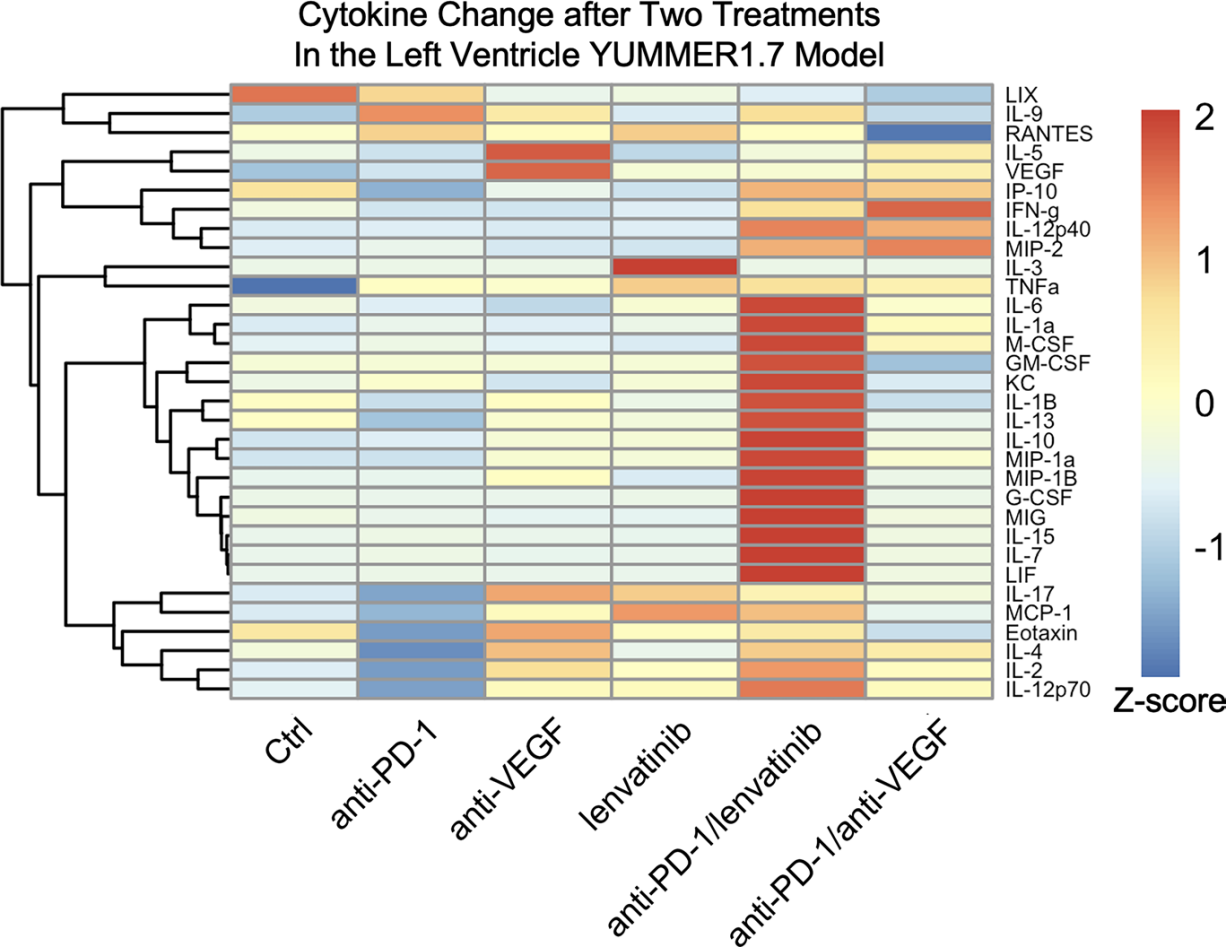
Combined antivascular-directed therapies and anti–PD-1 enhance antitumor inflammatory cytokines and chemokines. The change in cytokine levels after 2 i.p. treatments and/or a week of daily lenvatinib in the YUMMER1.7 LV melanoma model demonstrated enhanced macrophage activation and Th1 responses, particularly with anti–PD-1/lenvatinib. The T cell-stimulating cytokines IL-2, eotaxin, and IL-17 were elevated with anti-VEGF treatment. Cytokines associated with macrophage activation (G-CSF, MIP-1a, MIP-1B, M-CSF, and MIP-2) and T cell stimulation (IL-1a, MIG, IL-7, IL-15, IFN-γ, and IL-12p40), particularly Th1 cells (IL-2, TNF-α, IFN-γ, GM-CSF, IL-12p40, and IL-12p70) were increased with anti–PD-1/lenvatinib treatment even more so than with anti–PD-1/anti-VEGF treatment.

Early changes in plasma cytokines resulting from treatment were assessed. Anti-VEGF treatment resulted in higher circulating VEGF levels. The increase in VEGF due to anti-VEGF treatment was abrogated by combining it with anti–PD-1. Anti-VEGF treatment increased T cell-stimulating cytokines, including IL-2, eotaxin, and IL-17. Anti–PD-1/lenvatinib treatment resulted in increased cytokines/chemokines associated with macrophage activation (G-CSF, MIP-1a, MIP-1B, M-CSF, and MIP-2) and T cell stimulation (IL-1a, MIG, IL-7, IL-15, IFN-γ, and IL-12p40), particularly Th1 cells (IL-2, TNF-α, IFN-γ, GM-CSF, IL-12p40, and IL-12p70), more so than anti–PD-1/anti-VEGF treatment (Figure 4).

### Treatment with lenvatinib or anti-VEGF helps augment extravasation of immune cells in an in vitro model of the BBB.

**Figure 5:**
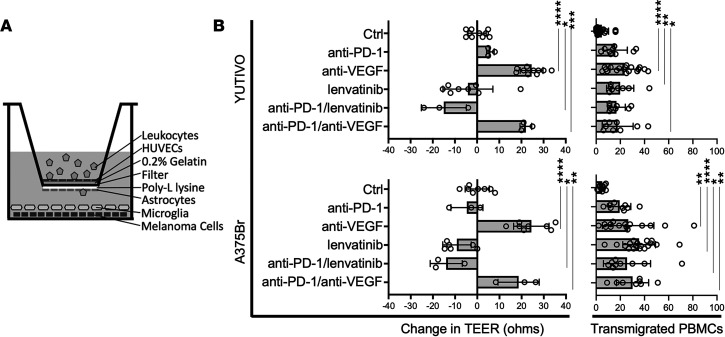
Treatment with anti-VEGF helps augment vascular normalization compared with lenvatinib, but both enhanced transendothelial migration of immune cells in an in vitro BBB model. (**A**) Schema of transwell assay containing leukocytes, HUVECs, astrocytes, microglia, and melanoma cells to recapitulate the BBB associated with BrMs. (**B**) Two human melanoma cultures were used: YUTIVO derived at Yale University from a melanoma BrM and the established cerebrotropic A375Br line. Both lines responded similarly in vitro to treatment. Compared with control (saline treated), treatment with anti-VEGF resulted in increased TEER, a marker for enhanced tightness of the BBB. Conversely, lenvatinib treatment resulted in decreased TEER. The number of activated PBMCs that migrated through the in vitro BBB after 24 hours of treatment and coculture were manually counted. Treatments containing anti-VEGF or lenvatinib, either alone or in combination with anti–PD-1, resulted in increased migration of immune cells. *P < 0.05, **P < 0.01, ***P < 0.001, and ****P < 0.0001 by 1-way ANOVA with correction for multiple comparisons.

The effect of anti–PD-1 alone or in combination with lenvatinib or anti-VEGF on the BBB was assessed in a transwell assay consisting of many of the cell types found in melanoma BrMs (Figure 5A). After 24 hours of drug treatment, transwells were carefully cut out and FITC^+^ PMBCs on the underside of the transwell were captured in 3 random photos per transwell and counted (Figure 5B and Supplemental Figure 5A). The in vitro assay was performed using YUTIVO, derived from a patient melanoma BrM, and A375Br, a cerebrotropic human melanoma cell line derived from A375 (32). Both melanoma cells responded similarly to drug treatment. Similar to our prior in vitro experiments without PBMCs, treatments containing anti-VEGF resulted in increased TEER from baseline, indicative of improved interendothelial tight junctions. However, treatments containing lenvatinib reduced TEER compared with control transwells. YUTIVO treatment with anti-VEGF alone or in combination with anti–PD-1 resulted in increased PBMC transwell migration (*P* < 0.0001 and *P* = 0.020, respectively). YUTIVO treatment with lenvatinib alone or in combination with anti–PD-1 also increased PBMC transmigration (*P* = 0.0066 and *P* = 0.055, respectively). Use of the second cerebrotropic melanoma cell line, A375Br, yielded similar results; treatment with anti-VEGF alone or in combination with anti–PD-1 resulted in increased PBMC transwell migration (*P* = 0.0077 and 0.0079, respectively). A375Br treatment with lenvatinib alone or in combination with anti–PD-1 also increased PBMC transmigration (*P* < 0.0001 and *P* = 0.038, respectively) (Figure 5B). TEER changes did not correlate with the amount of immune cell extravasation through the in vitro BBB (*r* = 0.28, *P* = 0.37) (Supplemental Figure 5B). The 1 μM lenvatinib dose used is well below the IC_50_ concentration of the cells used in our in vitro assay (Supplemental Tables 1 and 2).

### Combined lenvatinib or anti-VEGF therapy with anti–PD-1 can result in disparate tumor immune cell responses.

**Figure 6:**
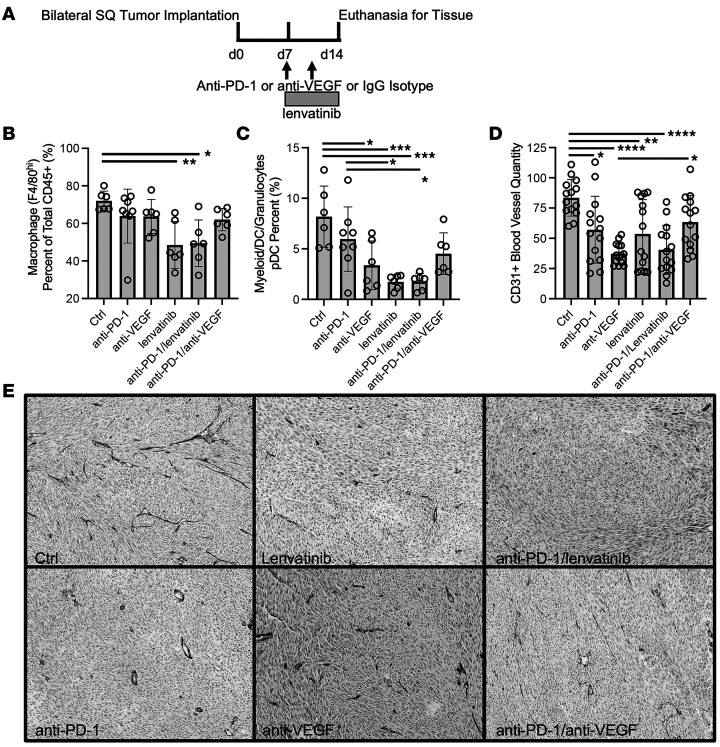
Flow cytometry analysis on YUMMER1.7 s.c. tumor-infiltrating leukocytes and IHC evaluation of tumor-associated blood vessels. (**A**) Experimental timeline for analysis of intratumoral changes. (**B**) Lenvatinib treatment resulted in decreased TAMs (defined as CD45^hi^/Ly6G^lo^/F4/80^hi^). (**C**) pDCs decreased with anti-VEGF and especially lenvatinib and anti–PD-1/lenvatinib therapy. (**D**) Quantification of tumor-associated blood vessels. Treatment with anti–PD-1, anti-VEGF, lenvatinib, and anti–PD-1/lenvatinib resulted in decreased CD31^+^ vessels when compared with control. (**E**) Representative photos of YUMMER1.7 tumors stained with anti-CD31 to highlight blood vessels. Photos taken on 10× magnification. *P < 0.05, **P < 0.01, ***P < 0.001, and ****P < 0.0001 by 2-way ANOVA with correction for multiple comparisons.

Mice injected with YUMMER1.7 melanoma cells into the LV developed multiple small intracranial metastases (Supplemental Figure 4C). Given the size of these micrometastases, it was not feasible to evaluate distinct intratumoral-immune subsets. As an alternative, animals were injected with YUMMER1.7 cells s.c. into both flanks, treated for 1 week, and tumor-infiltrating immune cells assayed by flow cytometry in 4 panels focused on macrophage, myeloid/DC/granulocyte, CD3 T cell, and CD8 memory T cell populations (Figure 6A). Distribution of cell subtypes as a proportion of the total population within each panel is found in Supplemental Figure 6, A, C, and D.

After 1 week of treatment, with an average total tumor volume of 277 mm^3^ ± 149 (range 15.33–537 mm^3^), tumors were subjected to flow cytometry analysis using a minimum of 3 animals per group. Lenvatinib alone or with anti–PD-1 resulted in decreased TAMs (defined as CD45^hi^/Ly6G^lo^/F480^hi^) compared with control (*P* = 0.0078 and 0.012, respectively) (Figure 6B). We did not detect statistically significant differences between treatment groups for M1- or M2-polarized macrophages (data not shown). Lenvatinib was also associated with increased tumor infiltration of neutrophils (PMNs; defined as CD45^hi^/Ly6G^hi^) compared with control or anti–PD-1 (*P* = 0.015 and 0.006, respectively), which was consistent histologically with the increased necrosis evident in the treated animals (*P* = 0.015), as shown by Yee et al. (33) (Supplemental Figure 6B). We found decreased plasmacytoid DCs (pDCs) (defined as CD45^hi^/Ly6G^lo^/CD19^hi^/CD172^hi^) with either lenvatinib or anti-VEGF compared with control (*P* = 0.0004 with lenvatinib; *P* = 0.012 with anti-VEGF; *P* = 0.0004 with anti–PD-1/lenvatinib; and *P* = 0.092 with anti–PD-1/anti-VEGF) (Figure 6C).

Surprisingly, there were no significant differences in major T cell subpopulations, including CD4 and CD8 subsets (Supplemental Figure 6C) and Treg cells (defined as CD3^hi^/CD4^hi^/Foxp3^hi^ cells) in the different treatment cohorts. Central memory (defined as CD3^hi^/CD8^hi^/CCR7^hi^/CD44^hi^ cells), effector memory (defined as CD3^hi^/CD8^hi^/CCR7^lo^/CD44^hi^ cells), and tissue-resident memory cells (defined as CD3^hi^/CD8^hi^/CD69^hi^/CD103^hi^) were not significantly altered with any of the treatment regimens (Supplemental Figure 6D). Treatment with anti–PD-1 increased the number of PD-1 negative cells that were detected in central memory populations. Interestingly, lenvatinib or anti–PD-1/lenvatinib also increased the population of PD-1 low central memory cells compared with control (*P* = 0.0089 and *P* < 0.0001, respectively) (Supplemental Figure 6E). Furthermore, lenvatinib or anti–PD-1/lenvatinib treatment resulted in increased PD-1 low tissue resident memory T cells negative for CD103 compared with controls (*P* = 0.023 and *P* < 0.0001, respectively) (Supplemental Figure 6F). CD103 negative tissue resident memory T cells have higher motility and transition to CD103 positive cells with TGF-β stimulation, which promotes their survival and retention in tissue (34). Anti-VEGF alone did not increase this population of cells, whereas anti–PD-1/anti-VEGF increased this memory population compared with control (*P* = 0.97 and 0.016, respectively).

To validate the findings from flow cytometry, s.c. and brain metastatic YUMMER1.7 tumors were stained with CD3 and CD8. We confirmed the lack of difference in CD3 and CD8 T cell populations between treatment groups (Supplemental Figure 7, A, B, D, and E). A CD163 Ab was used to identify macrophages, as more common macrophage markers CD68 and F4/80 had high background and coexpression by YUMMER1.7 cells. No differences were detected in CD163 populations, which was consistent with our flow cytometry data indicating a lack of difference in M2 populations between treatment groups (Supplemental Figure 7, C and F).

### Both lenvatinib and anti-VEGF result in decreases in tumor vessel count.

CD31 Ab was used to stain for tumor-associated blood vessels. Similar changes were noted for intratumoral and peritumoral vessels, so the data were combined for representation (Figure 6E and Supplemental Figure 4G). The number of CD31^+^ vessels was decreased with anti–PD-1, anti-VEGF, lenvatinib, and anti–PD-1/lenvatinib treatment compared with control (*P* = 0.020, < 0.0001, = 0.0054, and < 0.0001, respectively). Anti–PD-1/VEGF was almost as effective as anti–PD-1/anti-lenvatinib in decreasing the number of tumor-associated blood vessels (*P* = 0.064) (Figure 6D). Similar differences were seen qualitatively for intracranial tumor-associated vessels (Supplemental Figure 4G).

## Discussion

Our work provides what we believe to be novel and enhanced understanding of the biology behind angiogenesis and anti–PD-1 cotargeting. Prior work has used lenvatinib and anti–PD-1 in less immunogenic models, followed animals for less than 20 days, and did not assess memory responses (24, 35). We used YUMMER1.7 melanoma cells in our experiments as it is a well-documented, reproducible, more immune-sensitive model that harbors similar driver mutations to patient melanomas. The YUMMER1.7 model has well-established growth kinetics on ICIs and forms BrMs with LV injection (36). This study provides insights into the disparate mechanisms of antitumor immunity and effects on tumor-associated blood vessels, comparing anti-VEGF and anti-VEGFR targeting in extracranial and metastatic melanoma models, including in BrMs, which has not been reported.

We demonstrated that VEGFR and PD-1 cotargeting enhanced antitumor activity in melanoma and mice with complete regression of tumor had prolonged antitumor memory responses. Other VEGFRis may provide similar clinical benefit when combined with anti–PD-1 (37). While we described lenvatinib as mainly a pan-VEGFRi, it inhibits multiple receptor tyrosine kinases, and its effect on tumor angiogenesis may not be solely VEGFR mediated. FGFRs also promote tumor-associated angiogenesis; it is possible that dual targeting of VEGFRs and FGFRs augments anti-angiogenesis (20, 21). Treatments in our animal cohorts were done simultaneously — we did not evaluate the sequencing of therapies, such as starting with anti–PD-1 monotherapy and then adding an anti-VEGF/R. Therefore, we cannot address how the timing or sequence of these therapies impacts outcomes. We showed that lenvatinib and anti-VEGF decreased tumor-associated CD31^+^ vessels, either alone or in combination with anti–PD-1 compared with anti-VEGF. Therefore, for the purpose of reducing tumor-associated angiogenesis, lenvatinib may be a more biologically active treatment than anti-VEGF when combined with anti–PD-1. We believe lenvatinib is a potent inhibitor of tumor angiogenesis, as treatment-related hypertension is associated with response and thought to be caused by decreased nitric oxide production and defective vascular endothelial function (38). Additionally, lenvatinib’s secondary antitumor effect is its direct melanoma cytotoxicity, which was not VEGFR-2 dependent and alternatively could be due to effects on FGFRs, PDGFRα, KIT, or RET. Further studies will be needed to elucidate the exact mechanism of direct tumor cytotoxicity. Histologically, we observed that lenvatinib-treated s.c. YUMMER1.7 melanomas also had increased necrosis compared with anti-VEGF, which may be a direct effect of cytotoxicity and/or due to decreased tumor-associated blood vessels.

We demonstrated that ICIs and angiogenesis inhibitors in combination are more active against melanoma through modification of innate immunity and enhanced Th1 responses. We showed that lenvatinib decreased TAMs compared with anti-VEGF, results consistent with the literature (24). Interestingly, anti-VEGF treatment increased circulating VEGF levels, likely as a feedback mechanism to its peripheral inhibition or as a result of VEGF dissociation from the drug during measurement (39). Further evaluation of myeloid, DC, and granulocyte populations revealed that both lenvatinib and anti-VEGF decreased the number of infiltrating pDCs. Increased pDC infiltration has been associated with poor outcomes in several cancers, including melanoma, where pDCs contribute to immune evasion via high indoleamine 2,3-dioxygenase (IDO) and phospho-STAT3 expression (40). Thus, the reduction in pDCs with lenvatinib and anti-VEGF treatment could indicate a reversal of immune suppression within the TME. Of note, lenvatinib increased the infiltrating neutrophils, but we hypothesize that this effect is a byproduct of tumor necrosis.

In YUMMER1.7 tumors, no differences in the percentages of T cells or CD4 and CD8 subsets were identified across all treatment groups. However, we found early changes in PD-1^lo^ T memory populations. Zelba et *a*l showed that anti–PD-1 treatment competitively impedes the binding of additional PD-1 Abs, such as those used in flow cytometry (41). Therefore, anti-PD-1–containing treatments resulted in low detection of PD-1^hi^ memory T cells and increased the proportion of PD-1^lo^ memory T cells. The data on PD-1^hi^ versus PD-1^lo^ subpopulations are difficult to interpret given these technical limitations. Interestingly, lenvatinib monotherapy significantly increased PD-1^lo^ central memory T cells and a subpopulation of tissue resident memory cells defined as CD69^hi^/CD103^lo^. This suggested that lenvatinib is capable of alleviating part of the immune suppression in the TME. This effect was not seen with anti-VEGF alone. These results may help explain the in vivo superiority of lenvatinib compared with anti-VEGF in s.c. YUMMER1.7 tumors. The CD69^hi^/CD103^lo^ memory T cell population has been previously described and can emerge due to a lack of TGF-β signaling (42). The absence of greater differences in the memory populations could be due to early euthanasia after only a week of treatment. While this time point may ideally detect innate immunity changes, it may be limited in detecting memory changes. It would be technically challenging to assess immune differences, particularly in the anti–PD-1/lenvatinib treatment group by delaying tumor harvesting due to rapid tumor regression. All animals with fully regressed tumors demonstrated robust antitumor memory, suggesting durable responses.

Flow analysis of tumor-infiltrating leukocytes was limited to the s.c. melanoma model, as LV-injected animals developed small BrMs that were only sufficient for IHC. Alternate technologies and models will be needed to assess immune responses in BrMs, which is the focus of future research. Given the limitations in profiling immune infiltration and treatment on the BBB, we built upon previously established in vitro BBB models to better study treatment effects in the brain microenvironment. We showed that despite causing loss of endothelial barrier function, lenvatinib still promoted PBMC transmigration as efficiently as anti-VEGF treatment. This could suggest that lenvatinib may have additional immune modulating effects that are not solely based on its effect on the endothelium, an observation that has been previously described by Torrens et al. (43). Prior studies have indicated vascular normalization promotes leukocyte transmigration, but our studies demonstrated that this is the not the sole mechanism (44). Future studies will focus on the exact mechanism promoting transendothelial migration of immune cells as a result of lenvatinib.

In addition to providing data on leukocyte migration, our in vitro BBB provided insights into perilesional edema in BrMs, as TEER has an indirect relationship with endothelial leakiness and edema (29). Increased TEER represents reduced leakiness/edema, and decreased TEER signifies leakiness/edema. We found that anti-VEGF–containing therapies increase TEER and therefore may quickly reduce edema. This mechanism has already been clinically utilized for the treatment of tumor-associated edema in patients intolerant to corticosteroids (45–47). Conversely, lenvatinib decreased TEER and may potentially worsen edema. Considering the clinical challenges associated with treating patients with perilesional edema and BrMs, clinical studies comparing VEGF and VEGFRis in this setting are warranted.

Given the promise of combined PD-1 and angiogenesis blockade, our group has been conducting a phase II trial of pembrolizumab and bevacizumab in patients with previously untreated melanoma and non–small cell lung cancer BrMs (NCT02681549). We recently launched an investigator-initiated phase II trial of pembrolizumab and lenvatinib in untreated melanoma and renal cell BrMs (NCT04955743). Both studies will involve the analysis of correlative tissue specimens, which will lend further insight and validation to our in vivo and in vitro models.

Our studies provided insights into the mechanism of action of lenvatinib and anti-VEGF with anti–PD-1. Both anti-angiogenic approaches demonstrated enhanced activity when combined with anti–PD-1 versus when used as monotherapy. Although both enhanced Th1 signaling, subtle differences were observed, which may be clinically relevant when selecting an individualized regimen. For example, anti–PD-1/lenvatinib may result in faster antitumor responses but may temporarily worsen edema. Conversely, while anti–PD-1/anti-VEGF has good efficacy and is superior to anti–PD-1 alone, anti-VEGF has minimal single-agent activity and is contraindicated with recent surgery. For BrMs, anti–PD-1/anti-VEGF may be the superior option — it was comparable to anti–PD-1/lenvatinib in the LV model, improved BBB vascular normalization, and may provide rapid edema reduction. However, these findings need to be confirmed in prospective, randomized trials. Nonetheless, the insights gained in this study showing different effects of targeting VEGF or its receptors in combination with PD-1 inhibitors may help inform patient selection to optimize treatment and outcomes for those with metastatic melanoma. Additionally, future studies in other models of other cancer types may help determine whether the effects observed in these studies are shared with other malignancies.

## Methods

### In vitro drug sensitivity studies

Melanoma cells were grown as previously described, routinely tested for mycoplasma, and their identity confirmed via short tandem repeat profiling or exome sequencing (29, 48). Viability was measured with the CellTiter-Glo assay; details are provided in the Supplemental Methods.

### In vitro BBB assay

BBB transwells were prepared using a modified protocol (29, 49). Details on the transwell setup can be found in the Supplemental Methods.

### In vivo melanoma models

#### Subcutaneous model.

3 x 10^5^ YUMMER1.7 (gift from Marcus Bosenberg, Yale University; RRID:CVCL_A2AX) or B16-F10 (ATCC, catalog CRL-6475; RRID:CVCL_0159) cells were injected into the flanks of 9- to 11-week-old C57Bl6 male mice. Mice were given i.p. injections of 5 mg/kg anti-VEGF (clone G6-31, Absolute Antibody, catalog 1022-2.0) (50), 10 mg/kg anti–PD-1 (Bio X Cell, catalog BE0146; RRID:AB_10949053) (51), or 10 mg/kg isotype control (Bio X Cell, catalog BE0089; RRID:AB_1107769) twice weekly; drug combinations were dosed on the same day. Animals were given lenvatinib 10 mg/kg daily by oral gavage. Tumor volumes were calculated as previously described (36). For full details, see the Supplemental Methods.

#### LV model.

To test the intracranial efficacy of these drug combinations, YUMMER1.7 cells were injected into the LV of C57Bl6 mice, and growth was monitored by luminescence imaging. Treatment was administered on the same schedule as in the s.c. model but started on day 3 following LV injection, as this is a more aggressive model. For full details, see the Supplemental Methods.

### Flow cytometry

For full details, see the Supplemental Methods. In brief, primary-conjugated Abs were used to stain the cells for 30 minutes at 4°C in the dark (Supplemental Table 4). Data was analyzed using FlowJo (version 10.7; RRID:SCR_008520), and representative gating strategies can be found in Supplemental Figures 8–11.

### Histology

Tumors and brain were collected, formalin fixed, paraffin embedded, and stained with the ImmPRESS HRP Horse anti-rabbit IgG PLUS Polymer Kit (Vector Labs, catalog MP-7801). See Supplemental Methods for full details.

### Cytokine/chemokine profiling

Plasma from YUMMER1.7 LV-injected mice were collected at baseline and after 2 doses of IP treatment. Samples from 10 animals in each treatment cohort were pooled, and the relative changes in cytokine expression were analyzed using Eve Technologies’ Murine Cytokine Array/Chemokine Array 31-Plex (catalog MD31). Cytokine levels were normalized using standard reference levels per the company’s protocol. Results were analyzed in RStudio (version 1.4.1103; RRID:SCR_000432). A heatmap was generated using the pheatmap package in R (RRID:SCR_016418).

### Statistics

IC_50_, in vitro BBB, and flow cytometry data were analyzed using GraphPad Prism (version 8; RRID:SCR_002798). A 1-way or 2-way ANOVA was utilized for multivariate analysis with correction for multiple comparisons. Survival analysis was done using log-rank testing. *P* values less than 0.05 were considered significant.

### Study approval

Human melanomas were collected in accordance with the Declaration of Helsinki guidelines and approved by the Yale University IRB (Human Investigations Committee approval 0609001869) after providing written informed consent. All animal studies were performed in accordance with approved Yale IACUC protocols.

## Author contributions

TTT, JC, LZ, DS, DD, VLC, and VO conducted experiments. TTT, LBJ, and HMK conceptualized the study and designed the experiments. TTT, JC, VO, SAW, KO, LBJ, and HMK wrote and edited the manuscript.

## Supplementary Material

Supplemental data

## Version 1 02/23/2023

In-Press Preview

## Version 2 04/10/2023

Electronic publication

## References

[1] Cagney DN (2017). Incidence and prognosis of patients with brain metastases at diagnosis of systemic malignancy: a population-based study. Neuro Oncol.

[2] Tran TT (2019). Complications associated with immunotherapy for brain metastases. Curr Opin Neurol.

[3] Larkin J (2019). Five-year survival with combined nivolumab and ipilimumab in advanced melanoma. N Engl J Med.

[4] Motzer R (2021). Lenvatinib plus pembrolizumab or everolimus for advanced renal cell carcinoma. N Engl J Med.

[5] Makker V (2019). Lenvatinib plus pembrolizumab in patients with advanced endometrial cancer: an interim analysis of a multicentre, open-label, single-arm, phase 2 trial. Lancet Oncol.

[6] Lee WS (2020). Combination of anti-angiogenic therapy and immune checkpoint blockade normalizes vascular-immune crosstalk to potentiate cancer immunity. Exp Mol Med.

[7] Hodi FS (2014). Bevacizumab plus ipilimumab in patients with metastatic melanoma. Cancer Immunol Res.

[8] Ott PA (2015). Inhibition of immune checkpoints and vascular endothelial growth factor as combination therapy for metastatic melanoma: an overview of rationale, preclinical evidence, and initial clinical data. Front Oncol.

[9] Ohm JE, Carbone DP (2001). VEGF as a mediator of tumor-associated immunodeficiency. Immunol Res.

[10] Oyama T (1998). Vascular endothelial growth factor affects dendritic cell maturation through the inhibition of nuclear factor-kappa B activation in hemopoietic progenitor cells. J Immunol.

[11] Vanneman M, Dranoff G (2012). Combining immunotherapy and targeted therapies in cancer treatment. Nat Rev Cancer.

[12] Shrimali RK (2010). Antiangiogenic agents can increase lymphocyte infiltration into tumor and enhance the effectiveness of adoptive immunotherapy of cancer. Cancer Res.

[13] Huang H (2015). VEGF suppresses T-lymphocyte infiltration in the tumor microenvironment through inhibition of NF-κB-induced endothelial activation. FASEB J.

[14] Gabrilovich DI (1999). Antibodies to vascular endothelial growth factor enhance the efficacy of cancer immunotherapy by improving endogenous dendritic cell function. Clin Cancer Res.

[15] Dirkx AE (2006). Anti-angiogenesis therapy can overcome endothelial cell anergy and promote leukocyte-endothelium interactions and infiltration in tumors. FASEB J.

[16] Dineen SP (2008). Vascular endothelial growth factor receptor 2 mediates macrophage infiltration into orthotopic pancreatic tumors in mice. Cancer Res.

[17] Adamcic U (2012). The effect of bevacizumab on human malignant melanoma cells with functional VEGF/VEGFR2 autocrine and intracrine signaling loops. Neoplasia.

[18] Suyama K, Iwase H (2018). Lenvatinib: a promising molecular targeted agent for multiple cancers. Cancer Control.

[19] Yamamoto Y (2014). Lenvatinib, an angiogenesis inhibitor targeting VEGFR/FGFR, shows broad antitumor activity in human tumor xenograft models associated with microvessel density and pericyte coverage. Vasc Cell.

[20] Cross MJ, Claesson-Welsh L (2001). FGF and VEGF function in angiogenesis: signalling pathways, biological responses and therapeutic inhibition. Trends Pharmacol Sci.

[21] Lieu C (2011). Beyond VEGF: inhibition of the fibroblast growth factor pathway and antiangiogenesis. Clin Cancer Res.

[22] Adachi Y (2022). Inhibition of FGFR reactivates IFNγ signaling in tumor cells to enhance the combined antitumor activity of lenvatinib with anti-PD-1 antibodies. Cancer Res.

[23] Yi C (2021). Lenvatinib targets FGF receptor 4 to enhance antitumor immune response of anti-programmed cell death-1 in HCC. Hepatology.

[24] Kato Y (2019). Lenvatinib plus anti-PD-1 antibody combination treatment activates CD8+ T cells through reduction of tumor-associated macrophage and activation of the interferon pathway. PLoS One.

[25] Arance AM (2021). Lenvatinib (len) plus pembrolizumab (pembro) for patients (pts) with advanced melanoma and confirmed progression on a PD-1 or PD-L1 inhibitor: updated findings of LEAP-004. J Clin Oncol.

[26] Zhuang H (2019). Bevacizumab treatment for radiation brain necrosis: mechanism, efficacy and issues. Mol Cancer.

[27] Halstead MR, Geocadin RG (2019). The medical management of cerebral edema: past, present, and future therapies. Neurotherapeutics.

[28] Molhoek KR (2011). VEGFR-2 expression in human melanoma: revised assessment. Int J Cancer.

[29] Tran TT (2019). Perilesional edema in brain metastases: potential causes and implications for treatment with immune therapy. J Immunother Cancer.

[30] Bos PD (2009). Genes that mediate breast cancer metastasis to the brain. Nature.

[31] Liston DR, Davis M (2017). Clinically relevant concentrations of anticancer drugs: a guide for nonclinical studies. Clin Cancer Res.

[32] Xie TX (2006). Activation of stat3 in human melanoma promotes brain metastasis. Cancer Res.

[33] Yee PP (2022). Temporal radiographic and histological study of necrosis development in a mouse glioblastoma model. Front Oncol.

[34] Qiu Z (2021). TGF-β: many paths to CD103^+^ CD8 T cell residency. Cells.

[35] Yu JW (2018). Tumor-immune profiling of murine syngeneic tumor models as a framework to guide mechanistic studies and predict therapy response in distinct tumor microenvironments. PLoS One.

[36] Wang J (2017). UV-induced somatic mutations elicit a functional T cell response in the YUMMER1.7 mouse melanoma model. Pigment Cell Melanoma Res.

[37] Ciciola P (2020). Combining immune checkpoint inhibitors with anti-angiogenic agents. J Clin Med.

[38] Cabanillas ME, Takahashi S (2019). Managing the adverse events associated with lenvatinib therapy in radioiodine-refractory differentiated thyroid cancer. Semin Oncol.

[39] Alidzanovic L (2016). The VEGF rise in blood of bevacizumab patients is not based on tumor escape but a host-blockade of VEGF clearance. Oncotarget.

[40] Monti M (2020). Human plasmacytoid dendritic cells and cutaneous melanoma. Cells.

[41] Zelba H (2018). Accurate quantification of T-cells expressing PD-1 in patients on anti-PD-1 immunotherapy. Cancer Immunol Immunother.

[42] Tokura Y (2020). Pathophysiology of skin resident memory T cells. Front Immunol.

[43] Torrens L (2021). Immunomodulatory effects of lenvatinib plus anti-programmed cell death protein 1 in mice and rationale for patient enrichment in hepatocellular carcinoma. Hepatology.

[44] Duru G (2020). A window of opportunity: targeting cancer endothelium to enhance immunotherapy. Front Immunol.

[45] Meng X (2017). Efficacy and safety of bevacizumab treatment for refractory brain edema: case report. Medicine (Baltimore).

[46] Bai X (2021). Efficacy of bevacizumab in the treatment of refractory brain edema of metastatic tumors from different sources. Neurol Res.

[47] Banks PD (2019). Bevacizumab as a steroid-sparing agent during immunotherapy for melanoma brain metastases: a case series. Health Sci Rep.

[48] Thumar J (2014). MEK targeting in N-RAS mutated metastatic melanoma. Mol Cancer.

[49] Jilaveanu LB (2015). PLEKHA5 as a biomarker and potential mediator of melanoma brain metastasis. Clin Cancer Res.

[50] Mordenti J (1999). Efficacy and concentration-response of murine anti-VEGF monoclonal antibody in tumor-bearing mice and extrapolation to humans. Toxicol Pathol.

[51] Bernardo M (2021). An experimental model of anti-PD-1 resistance exhibits activation of TGFβ and Notch pathways and is sensitive to local mRNA immunotherapy. Oncoimmunology.

