# Supplementary material for: Lenvatinib or anti-VEGF in combination with anti–PD-1 differentially augments antitumor activity in melanoma
Source: JCI Insight. 2023 Apr 10;8(7):e157347. doi: 10.1172/jci.insight.157347 (PMC10132152; doi:10.1172/jci.insight.157347)
Supplement: Supplemental data [file jciinsight-8-157347-s099.pdf]

Lenvatinib or Anti-VEGF in Combination with Anti-PD-1 Differentially Augments Anti-Tumor Activity in Melanoma

Authors and affiliations
Thuy T. Tran[1], Jasmine Caulfield[1], Lin Zhang[1], David Schoenfeld[1], Dijana Djureinovic[1], Veronica L. Chiang[1,2], Victor Oria[1], Sarah A. Weiss[1], Kelly Olino[1,3], Lucia B. Jilaveanu[1*], Harriet M. Kluger[1*]

[1]Yale School of Medicine and Yale Cancer Center, New Haven, CT 06510, USA
[2]Yale School of Medicine, Department of Neurosurgery, New Haven, CT 06510, USA
[3]Yale School of Medicine, Department of Surgery, New Haven, CT 06510, USA
* These authors contributed equally to this work

Supplemental Methods:

In vitro Drug Sensitivity Studies
Lenvatinib (LC Laboratories Cat# L-5400) and anti-VEGF (Selleck Chemicals Cat# A2006) were added at specified doses to 2,000 cells plated in serum-free Opti-MEM on a 96-well plate. Assays were conducted in quadruplicate. After 72 hours of incubation CellTiter-Glo (Promega Cat# G9683) was used to determine the relative amounts of viable cells, and luminescence was measured.

In vitro blood-brain barrier assay
Briefly, the outer membrane of 3-micron pore PET membrane transwells (Falcon Cat# 353492) for permeability studies or FluroBlok cell culture inserts (Corning Cat# 351151) for leukocyte transmigration studies were coated with 1 ug/mL of poly-L-lysine (ScienCell Cat# 0413) overnight at 37C. The following day, the inner membrane was coated with 0.2% gelatin (Sigma Cat# G1393) at 37C for 30 minutes. Residual coating solutions were removed, and the transwells inverted. 1e5 E6/E7/hTERT immortalized astrocytes (gift from Dr. Timothy Chan, Cleveland Clinic, Cleveland, OH) were seeded onto the outer transwell and fed with additional complete astrocyte media (DMEM [Gibco Cat# 11965092] supplemented with 10% FBS [Gibco Cat# A5256801]) every 15 minutes for 4 hours. The transwells were gently lowered into a 24-well plate containing complete endothelial cell growth media (ECM, ScienCell Cat# 1001), and 5e4 primary human umbilical vein endothelial cells (HUVECs, ScienCell Cat# 8000) in ECM were added to the inner transwell. HUVECs were allowed to form tight junctions over the next 3 days, undisturbed.

For the permeability assay, treatment with either 1 μM of lenvatinib, 1 mg/mL of anti-VEGF (Mvasi, bevacizumab-awwb), or 10 ug/mL of anti-human PD-1 (Clone J116, Bio X Cell, Cat# BE0188, RRID:AB_10950318) was added. TEER was measured using STX2 chopstick electrodes (EVOM^2, World Precision Instruments) at baseline prior to treatment and after 24 hours of co-culture.

For the leukocyte transmigration assay, on day 2 of the transwell co-culture, 5e4 primary short-term melanoma brain metastasis cells were mixed 1:1 with HMC3 microglia (ATCC Cat# CRL-3304, RRID:CVCL_II76) and cultured overnight in a 24-well plate with ECM. PBMCs were collected from healthy donors using a Ficoll-Paque Plus (density 1.077; GE Healthcare Cat# 17144003) per manufacturer's protocol. Cells were washed twice with cold phosphate-buffered saline and stained with 15 μM of CellTracker Green 5-chloromethylfluorescein diacetate (Invitrogen Cat# C2925) per protocol. T cell stimulation was performed by resuspending PBMCs

in RPMI complete media (Gibco Cat# A1049101) at 1e6 cells/mL. Cells were added into 24-well culture plates pre-coated for 2 hours at 37C with 10 µL of CD3 UCHT1 antibody (BD Biosciences Cat# 550368, RRID:AB_393639). CD28 clone 28.2 antibody (BD Biosciences Cat# 556620, RRID:AB_396492) was added at 5 ug/mL to the PBMCs, and the plate was incubated overnight. On day 3, the transwell inserts containing attached primary HUVECs and E6/E7/hTERT immortalized astrocytes were lifted out of the 24-well plate using sterile forceps, and the media in the inner transwell was gently removed. The transwell was gently lowered into the plate containing melanoma cells and microglia. The CellTracker labeled and stimulated PBMCs were washed and resuspended in ECM containing 50 ug/mL of Texas Red-labeled BSA (Invitrogen Cat# A23017). Appropriate concentrations of drugs were added to respective transwells: 1 µM of lenvatinib (LC Laboratories), 1 mg/mL of anti-VEGF (Mvasi, bevacizumab-awwb), and 10 ug/mL of anti-human PD-1 (Clone J116, Bio X Cell). TEER was measured at baseline and after 24 hours of co-culture. After 24 hours, the transwells were washed gently with ice-cold PBS and fixed using 4% PFA at room temperature for 10 minutes. The transwells were gently washed twice with PBS and stained with DAPI (1:1000) (ThermoFisher Cat# 62248) for 30 minutes. After 2 additional washes with PBS, the transwells were cut out and mounted using Prolong Gold with DAPI (Invitrogen Cat# P36930). Three random photos from the outer surface of each transwell membrane was photographed on an Olympus BX41 scope at 10X magnification, and cells were manually counted. Conditions were tested in triplicate and repeated at least twice.

In vivo Melanoma Models

Subcutaneous Model

Melanoma cells were trypsinized, washed twice with ice-cold PBS, and $3x10^5$ cells in 100 µL of PBS were injected into the shaved flanks of wild-type C57Bl6 male mice (The Jackson Laboratory, RRID:IMSR_JAX:000664). Male mice were used to avoid sex-related rejection of cells. Tumors were palpable after 7 days when treatment began. Tumors were measured twice a week. YUMMER1.7 injected animals were treated for 31 days and then monitored. Animals that had complete regression of their tumor were re-challenged with 5e5 cells to test for anti-tumor memory responses. B16-F10 injected animals were continually treated until the last mouse had to be euthanized due to tumor size >1000 mm^3 or tumor ulceration. Blood was collected for plasma and PBMCs at day 7 prior to first treatment, at day 14, and upon euthanizing the animal. Tumor was collected at sacrifice for histology. A separate cohort of YUMMER1.7 injected animals was used for tumor flow cytometry analysis. These animals received bilateral injection of tumor cells to maximize tissue for analysis and 2 doses of treatment before being euthanized to collect blood and tumor for flow cytometry. Three animals were analyzed per treatment cohort. All cells were tested by the Yale Molecular Diagnostics Laboratory and negative for murine pathogens.

Left Ventricle Model

$1x10^5$ YUMMER1.7 cells in 100 µL of PBS was directly injected into the left ventricle of Nair treated, wild-type C57Bl6 8-week-old, male mice. YUMMER1.7 had previously been engineered to express luciferase. Animals were injected with luciferin and IVIS-imaged (PerkinElmer Lumina X5) immediately after LV injection to confirm systemic dissemination of cells. Animals were IVIS-imaged again on day 3-4 post injection. Only animals with detectable luminescence intracranially on day 3-4 were included for subsequent analysis. Intracranial luminescence signals were defined by regions of interest over the dorsal head; extracranial signal was defined by ventral regions of interest. Blood was collected at baseline prior to initiation of treatment, a week after initiation of treatment, and again upon sacrificing of the animal at the onset of any neurologic symptoms or weight loss >20% from baseline. Brains were resected for histology with an additional cohort of animals imaged via IVIS in vivo and ex vivo immediately prior to euthanasia.

Flow cytometry

After tumor resection, tissue was minced and digested in a 37C water bath using RPMI (Gibco) supplemented with 2% FBS (Gibco), 0.1 mg/mL collagenase (Sigma Cat# 10103578001), and 0.4 mg/mL DNase I (Roche Cat# 4716728001) for 30 minutes with intermittent agitation. Cells were filtered through a 70-micron cell strainer. After pelleting the cells in a centrifuge, the supernatant was removed, and 1 mL of RBC lysis buffer was added to the samples for 2 minutes. 9 mL of RPMI was added before spinning the cells down again. Standard flow cytometry staining was performed. $2x10^6$ cells each were used for staining. Cells were blocked using Fc block (1:100) for 20 minutes on ice. Cells were filtered through a 70-micron cell strainer prior to processing on a BD LSR II 5-UV (Yale Flow Cytometry Core). Gating strategies are depicted in **Supplemental Figures 8-11.**

Histology

For brain sections, metastases were identified by obtaining 5 serial slides (one of which was an H&E section) every 50 microns through the brain tissue. Sections with identifiable tumors based on H&E was utilized for subsequent stains. Paraffin was removed by heating slides in a 60C oven until soft, followed by two washes with xylene for 5 minutes, and rehydration in 2 washes each of 100% ethanol, 70% ethanol, and water. Citrate antigen retrieval was performed in a steamer for 20 minutes (for CD8 and CD31) or 30 minutes (for CD3 and CD163) followed by cooling to 45C and another wash in water for 5 minutes. The ImmPRESS HRP Horse anti-rabbit IgG PLUS Polymer Kit (Vector Labs) was utilized for subsequent staining with primary antibody incubation overnight at 4C in a humidified chamber. Slides were counter stained with hematoxylin for 15 seconds and bluing reagent for 30 seconds. Slides were then dehydrated in increasing alcohol gradients followed by xylene before mounting using Permount (Fisher Scientific Cat# SP15-100). Antibodies utilized are as follows: CD3 (clone SP7, Novus biologicals, 1:100, Cat# NB600-1441, RRID:AB_789102), CD8 (Cell Signaling, 1:200, Cat# 98941S, RRID:AB_2756376), CD163 (clone EPR19518, Abcam, 1:500, Cat# ab182422, RRID:AB_2753196), CD31 (clone D8V9E, Cell Signaling, 1:100, Cat# 77699S, RRID:AB_2722705). Six random photos were taken from each tissue at 10X magnification and represent intratumoral and peritumoral tissue. Positive cells were either manually counted or processed using the EBimage R package.

Supplemental Figures

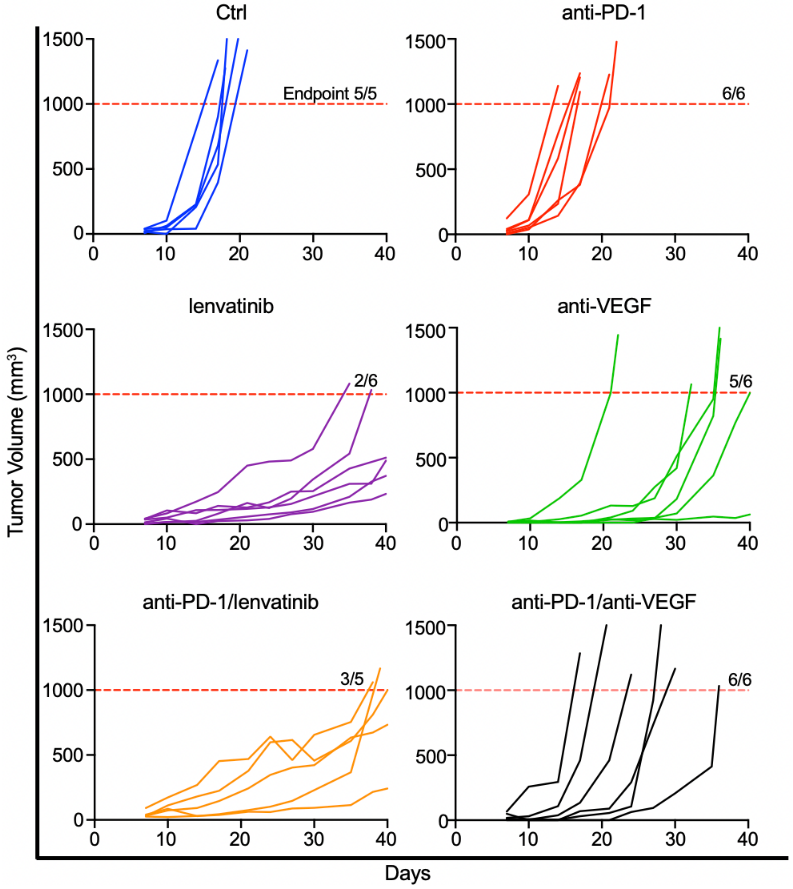

Supplemental Figure 1. B16-F10 melanoma tumor growth curves. Individual tumor volume measurements for animals subcutaneously injected with B16-F10 melanoma cells.

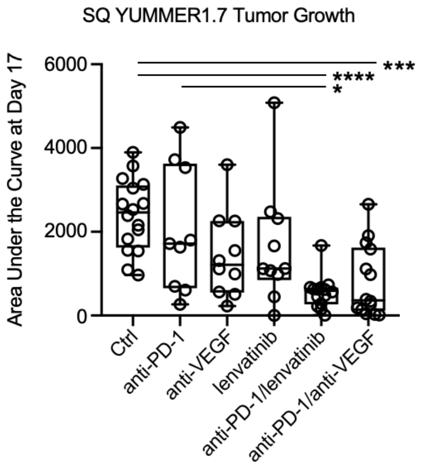

Supplemental Figure 2. Treatment with anti-PD-1 combined with either lenvatinib or anti-VEGF resulted in delayed tumor growth as early as day 17. Box and whisker plot indicating median and 25th to 75th percentiles of subcutaneous (SQ) tumor growth. Bars indicate the range. Circles represent individual data points. **P* < 0.05, ****P* < 0.001, and **** *P* < 0.0001.

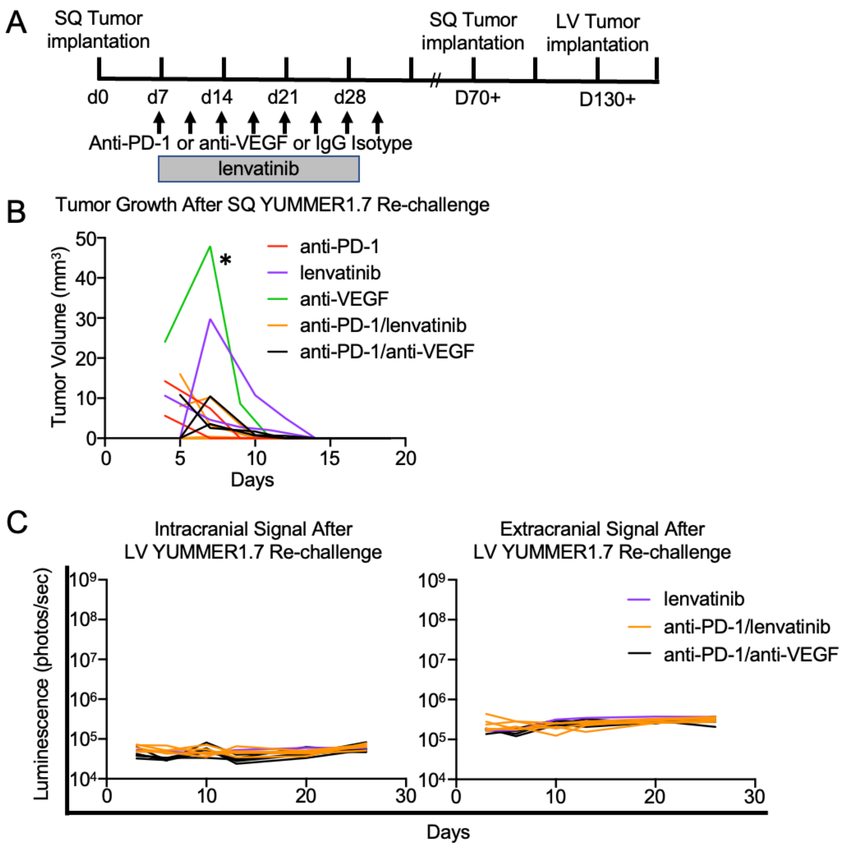

Supplemental Figure 3. Re-injection of YUMMER1.7 melanoma cells resulted in complete rejection of tumor and demonstrated robust anti-tumor memory responses. A. Experimental layout showing initial SQ injections of tumor followed by treatment, discontinuation of treatment after day 31, and re-injection beyond day 70 first via SQ then via left ventricle (LV) in animals who had full tumor regression. Re-injected animals were not retreated. **B.** SQ-injected animals were monitored for tumor growth until full regression recurred by day 15 post-injection. Of note, the anti-VEGF treatment animal (*) included never developed a tumor >20 mm^3 at the time of the original injection and was censored from prior studies. However, this animal was treated with anti-VEGF for the full duration of the initial experiment so was included in the re-challenge experiment. **C.** The same animals were subsequently injected with YUMMER1.7 cells into the left ventricle, and IVIS luminescence signal was measured for 4 weeks. No increase in luminescence was detected in the brain or body of these animals. Several animals did not tolerate LV injection; thus, fewer animals are represented in the LV-injected experiments. n = 1, 3, and 5 in lenvatinib, anti-PD-1/lenvatinib, and anti-PD-1/anti-VEGF groups, respectively.

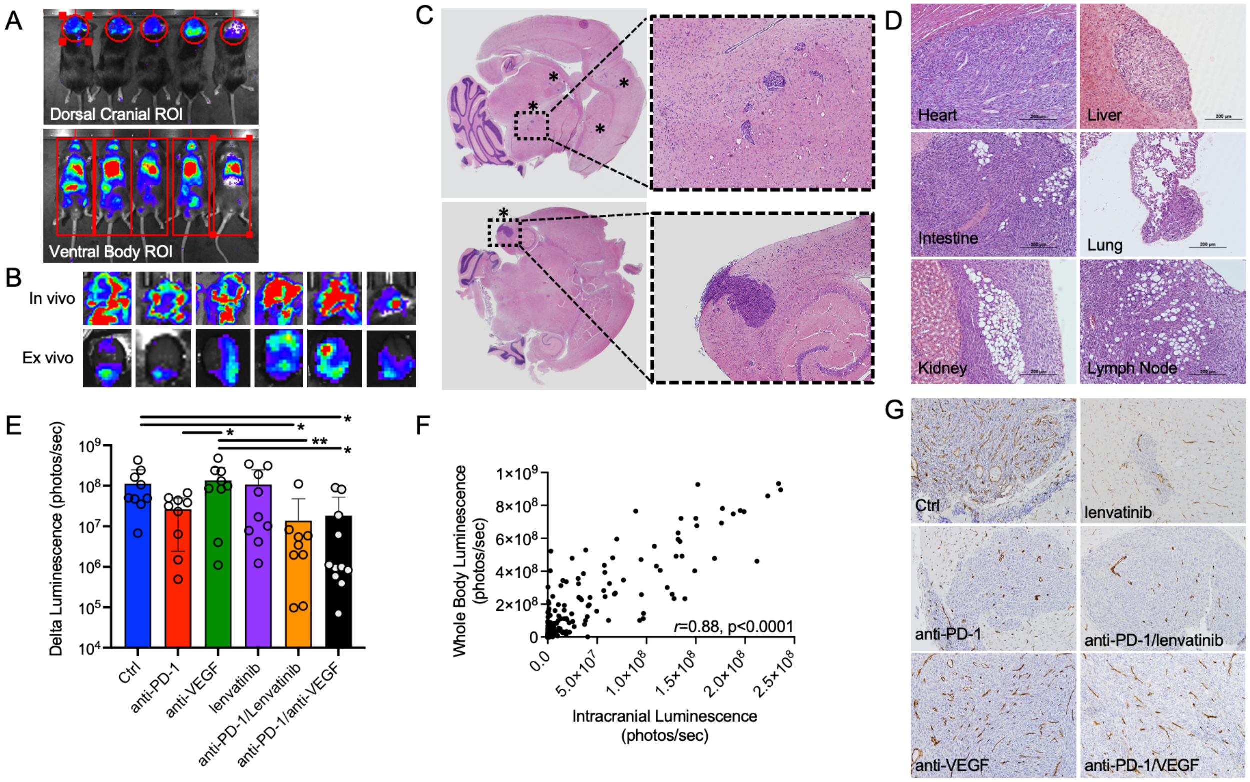

Supplemental Figure 4. Left ventricle injections of YUMMER1.7 model disseminated melanoma. A. Representative images depicting luminescence regions of interest (ROI) intracranially and whole body on day 5 post injection. **B.** Paired in vivo and immediate ex vivo images of brains demonstrating that higher signals in vivo likely represent extra parenchymal metastases. **C.** Representative brains stained with hematoxylin and eosin containing brain metastases (subset magnified at 10X). **D.** Representative 10X hematoxylin and eosin photos of additional organs of left ventricular injected animals demonstrating wide-spread metastases. **E.** Whole body, ventral luminescence signals from LV-injected animals demonstrated a significant decrease in tumor burden in animals treated with anti-PD-1/lenvatinib and anti-PD-1/VEGF compared to Ctrl (p=0.031 and 0.035, respectively) by day 16 post injection. *$P < 0.05$ and **$P < 0.01$. **F.** A strong correlation exists between whole body and intracranial luminescence for all animals and timepoints (Pearson's $r = 0.88$, $P < 0.0001$). **G.** Representative 10X CD31 staining (brown) of intracranial tumors.

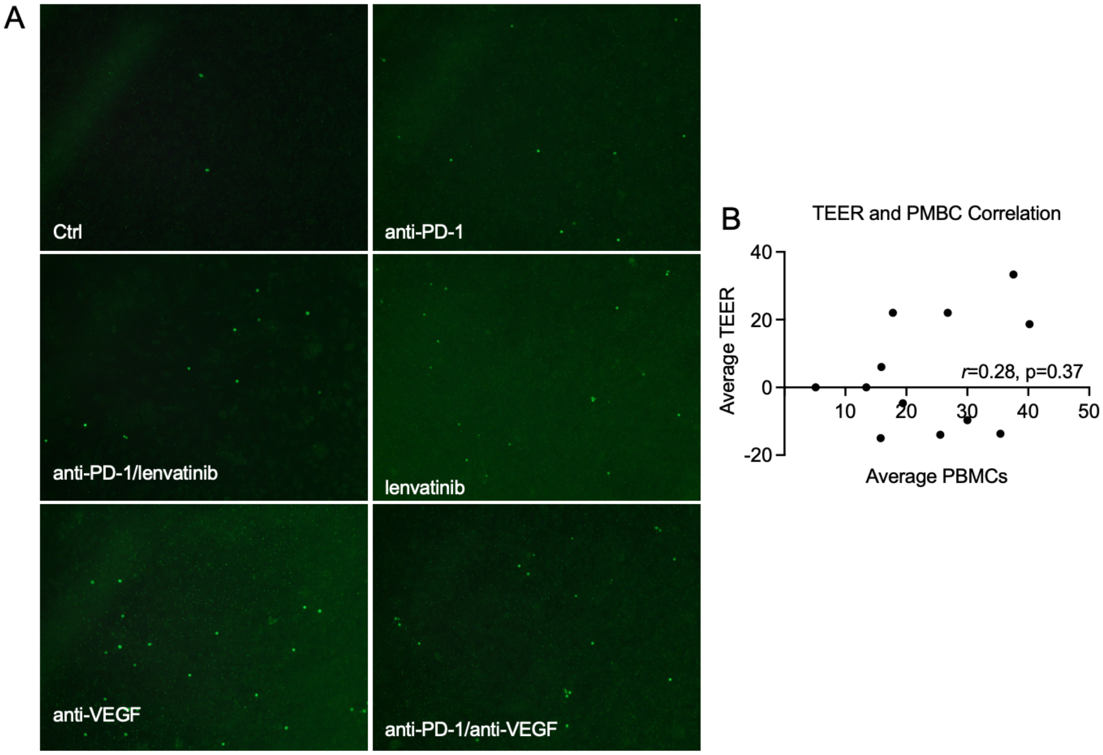

Supplemental Figure 5. Lenvatinib and anti-VEGF treatments enhance PBMC transmigration, which does not correlate with TEER changes. A. Representative photos of transmigrated PBMCs captured from the underside of FluoroBlok transwell membranes. PBMCs were FITC-labeled (green). **B.** TEER changes did not correlate with number of transmigrated PBMCs (r = 0.28, P = 0.37).

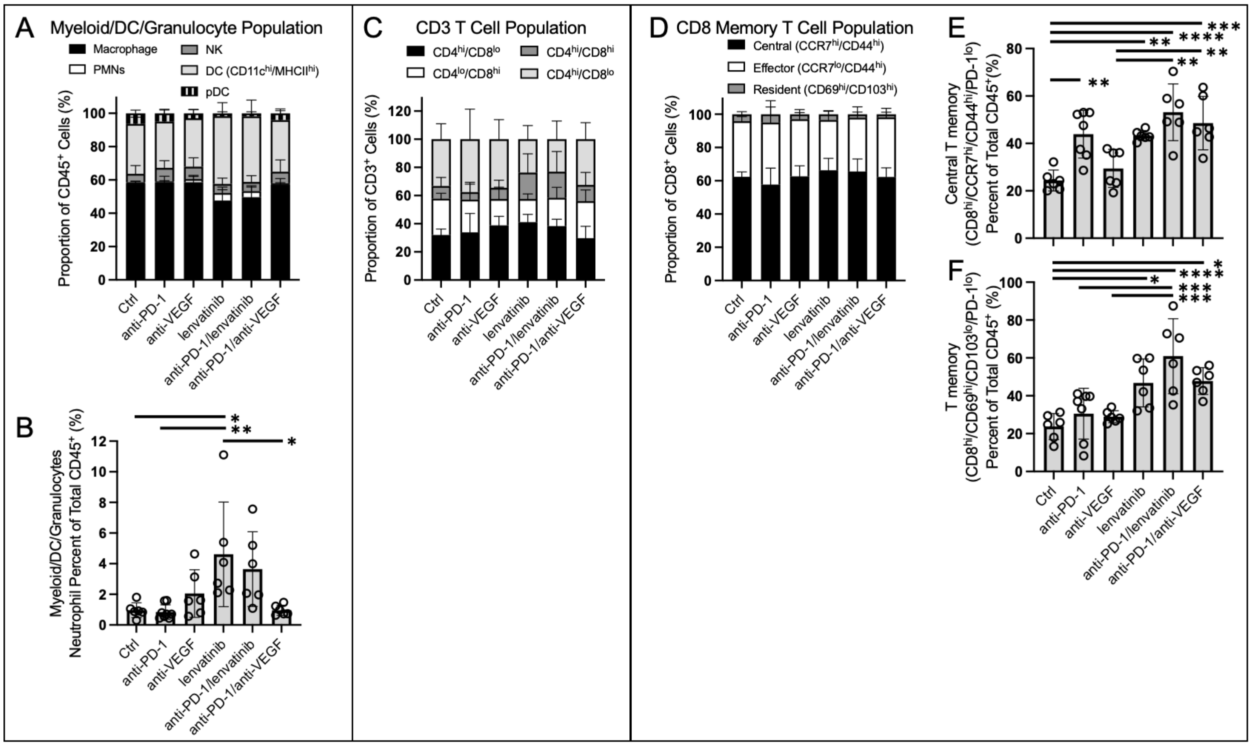

Supplemental Figure 6. Flow cytometry analysis of tumor immune infiltrating cells. A. Distribution of myeloid cells, dendritic cells, and granulocytes as a proportion of live, CD45 positive cells that were negative for CD3/CD19/TCR. **B.** Analysis of the myeloid cell, dendritic cell, and granulocyte populations revealed an increase in neutrophils with lenvatinib treatment. **C.** Distribution of CD3 positive T cells by CD4 and CD8 expression. **D.** Distribution of CD8 positive memory T cell subsets. **E.** Analysis of memory CD8 T cell populations revealed an increase in PD-1 low central T memory cells with anti-PD-1 or lenvatinib treatment. **F.** Anti-PD-1 with anti-VEGF or lenvatinib along with lenvatinib monotherapy increased the $CD103^{lo}/PD\text{-}1^{lo}$ subpopulation of memory T cells. $^{*}P < 0.05$, $^{**}P < 0.01$, $^{***}P < 0.001$, and $^{****}P < 0.0001$.

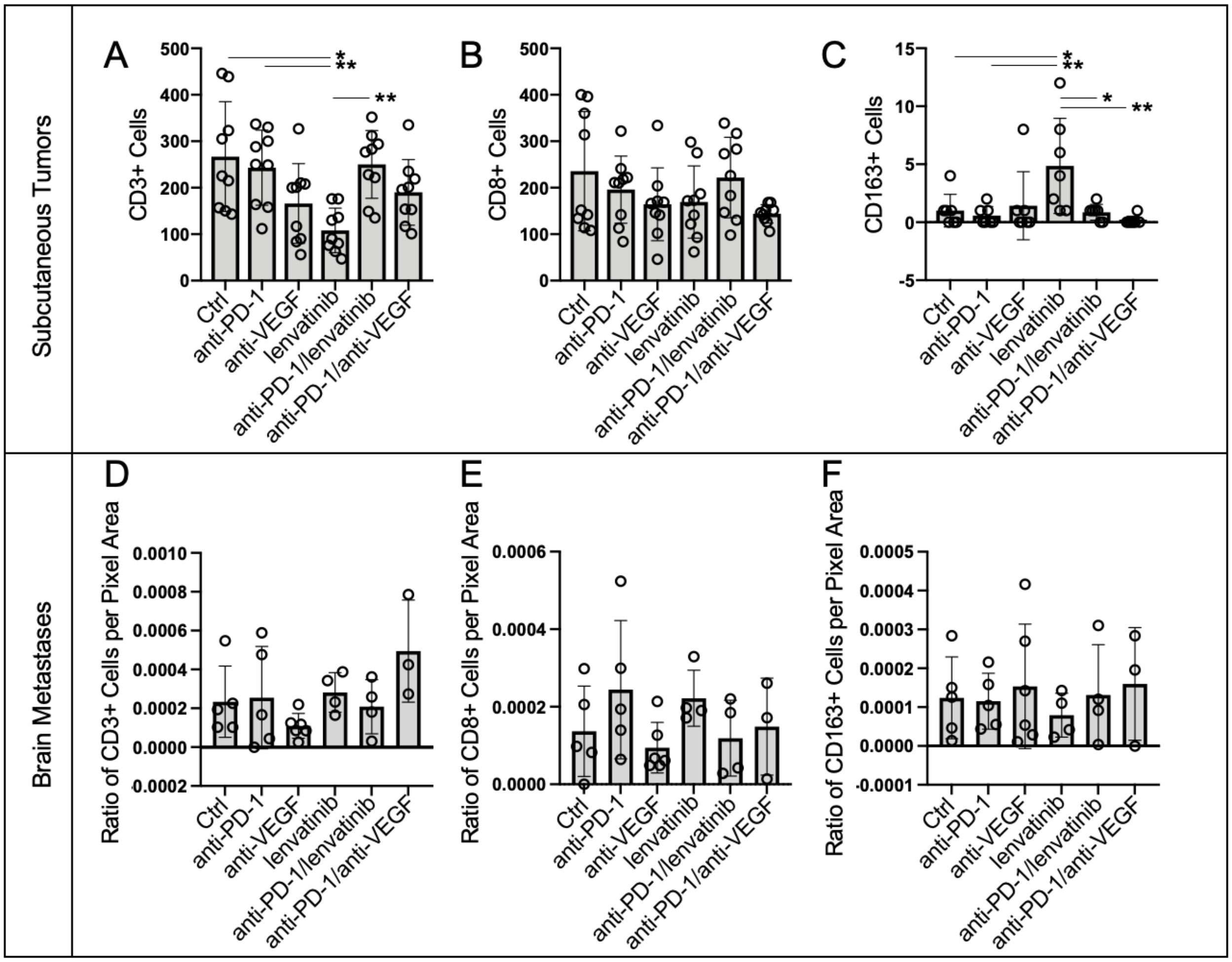

Supplemental Figure 7. Immunohistochemistry of treated YUMMER1.7 tumors. A. Number of intratumoral CD3 positive cells in subcutaneous YUMMER1.7 tumors. **B.** Number of intratumoral CD8 positive cells in subcutaneous YUMMER1.7 tumors. **C.** Number of intratumoral CD163 positive cells in subcutaneous YUMMER1.7 tumors. N=3 animals in each cohort. **D.** Number of intratumoral CD3 positive cells in YUMMER1.7 brain metastases. **E.** Number of intratumoral CD8 positive cells in YUMMER1.7 brain metastases. **F.** Number of intratumoral CD163 positive cells in YUMMER1.7 brain metastases. Each circle represents one animal brain. *$P < 0.05$, **$P < 0.01$, ***$P < 0.001$, and ****$P < 0.0001$

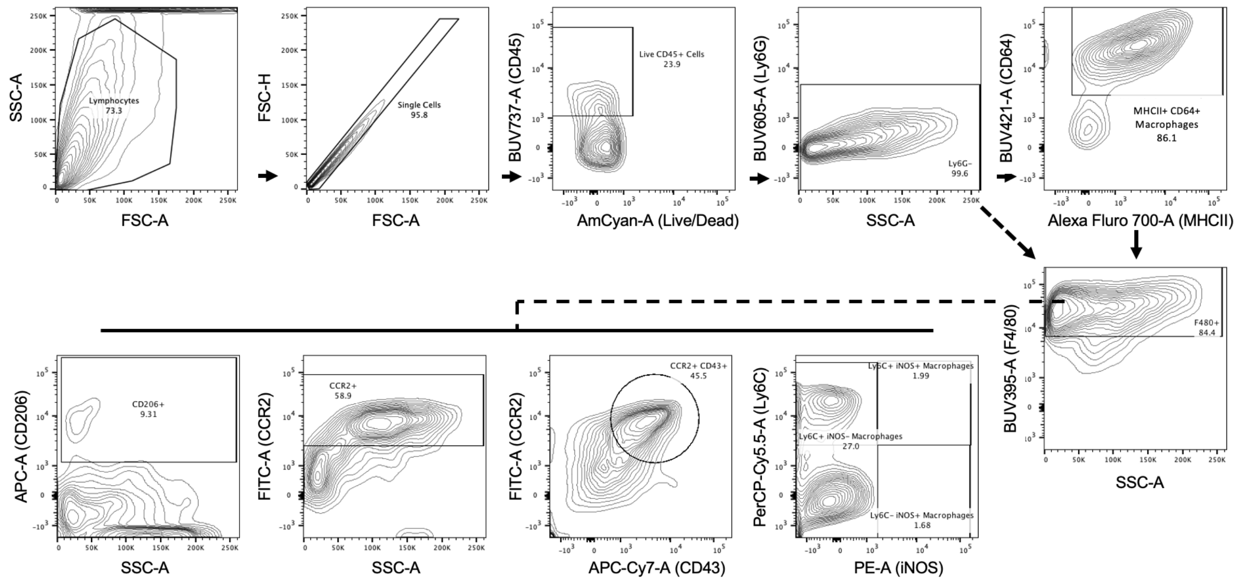

Supplemental Figure 8. Flow cytometry gating strategy for tumor immune infiltrating macrophage subsets. Example gating for macrophage populations. F4/80 positive macrophages were both assessed from the parent Ly6G- or MHCII+/CD64+ populations (dashed arrow versus solid arrow, respectively).

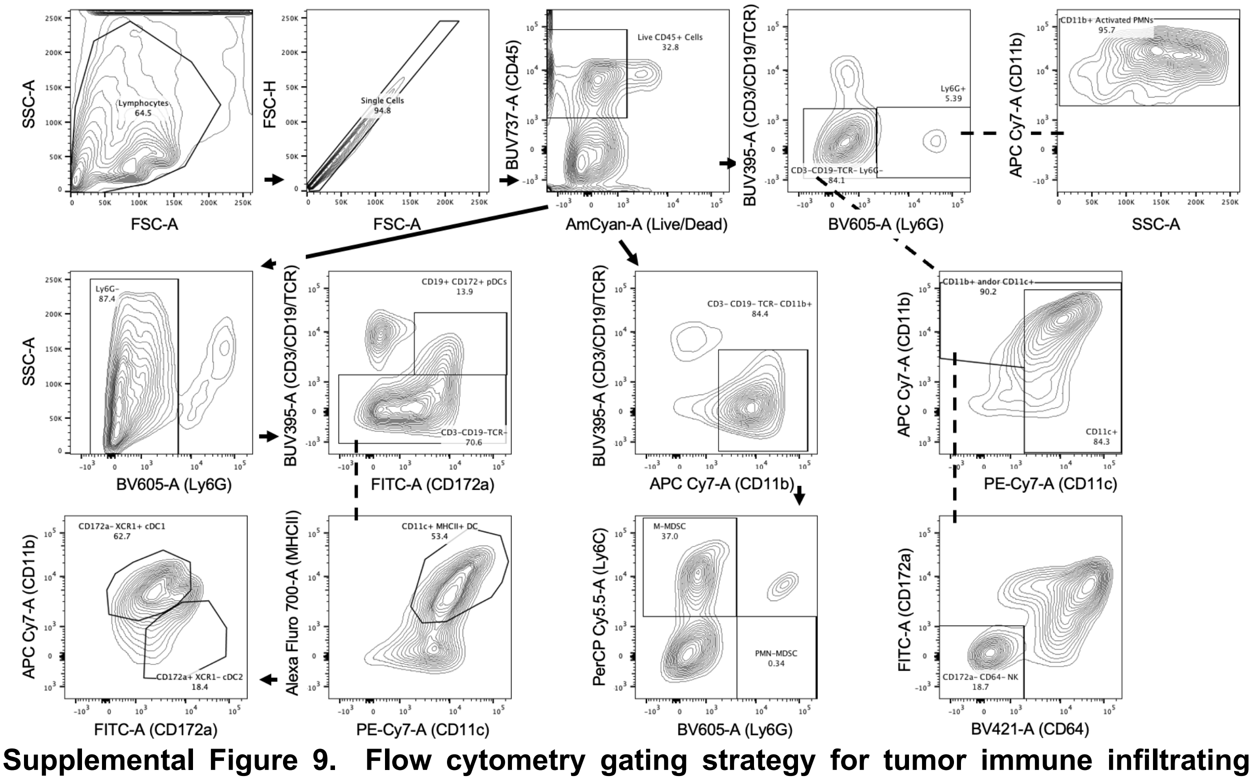

Supplemental Figure 9. Flow cytometry gating strategy for tumor immune infiltrating myeloid cell, dendritic cell, and granulocyte subsets. Example gating for myeloid cell, dendritic cell, and granulocyte populations.

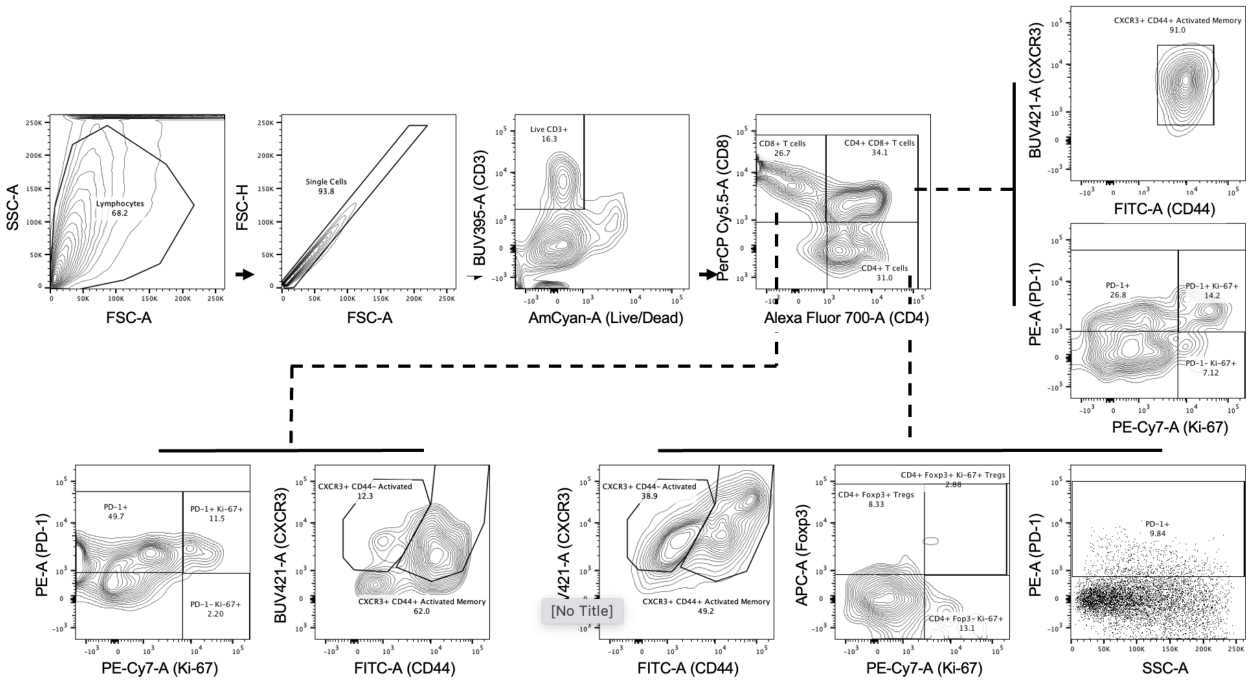

Supplemental Figure 10. Flow cytometry gating strategy for tumor immune infiltrating T cell subsets. Example gating for CD3+ T cell populations.

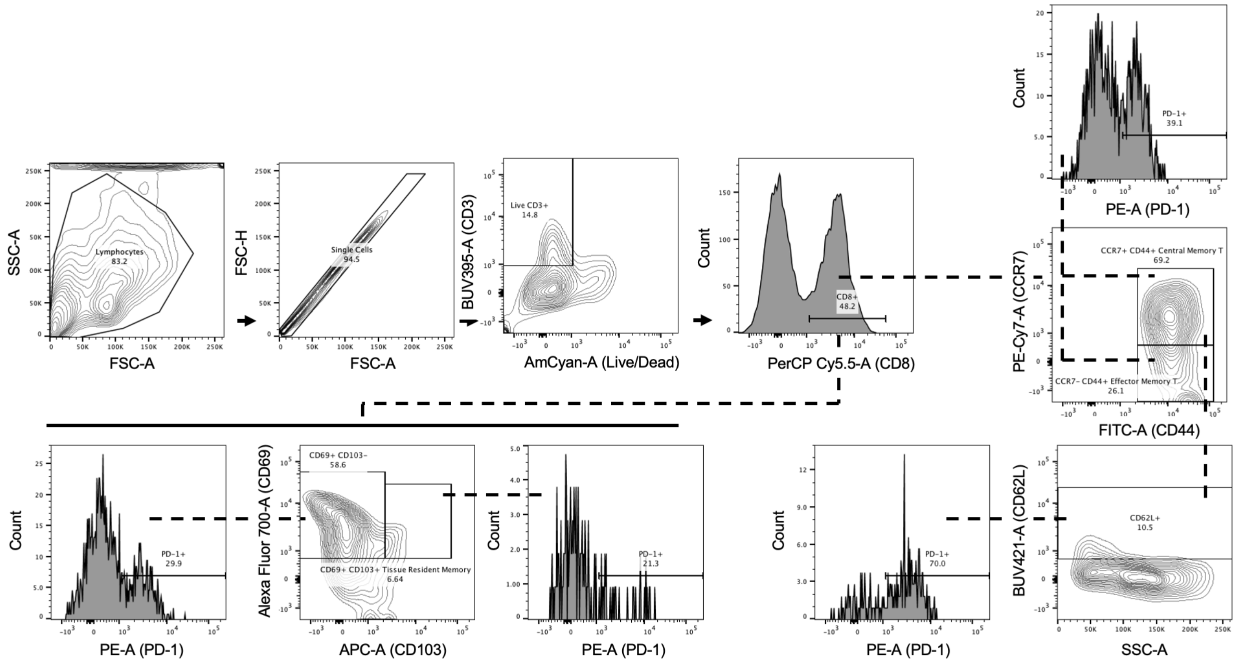

Supplemental Figure 11. Flow cytometry gating strategy for tumor immune infiltrating memory T cell subsets. Example gating for memory T cell populations.

Supplemental Tables

Supplemental Table 1.

Cell Culture	Metastatic Site	Lenvatinib IC_{50} (μM)*	Bevacizumab IC_{50} (μg/mL)*
YUVENA-M2	Intracranial	3.3	NR
YUMETRO	Intracranial	5.0	NR
YUTIVO	Intracranial	5.6	NR
YUTICA	Extracranial	6.1	NR
YUVON	Extracranial	7.0	NR
YUVENA	Intracranial	8.4	NR
YUGASP	Extracranial	10.1	NR
YUSIDRA	Intracranial	10.3	NR
YUSIK	Extracranial	10.6	NR
YUSIT	Extracranial	12.4	NR
YUTIAN	Intracranial	13.1	NR
A375Br	Intracranial	18.1	NR
YUKSI	Extracranial	28.5	NR
YUKRIN	Intracranial	NR	NR
YUCHER	Extracranial	NR	NR
YUAME	Extracranial	NR	NR
YUSIV	Extracranial	NR	NR
YUCOT	Extracranial	NR	NR

*Mean of 4 replicates. NR=Not reached (>100 μM lenvatinib or >300 μg/mL bevacizumab)

Human metastatic melanoma cultures from intracranial and extracranial metastases demonstrate superior direct cytotoxic activity of lenvatinib compared to bevacizumab. Short-term melanoma cultures derived from patients treated at Yale University are designated by the "YU" prefix. A375Br is a cerebrotropic daughter line derived from serial carotid injection and in vitro expansion of brain metastases in mice.

Supplemental Table 2.

Cell Culture	Tissue	Lenvatinib IC_{50} (μM)*
HUVEC	Endothelial Cell	16.1
E6/E7/hTERT Immortalized Astrocytes	Astrocytes	3.4
HMC3	Microglia	9.7

*Mean of 4 replicates.

Cytotoxicity of lenvatinib in cells constituting the in vitro BBB. IC_{50} data for the melanoma cells used can be found in Supplemental Table 1.

Supplemental Table 3.

Cell Culture	Tissue	Lenvatinib IC_{50} (μM)*	Bevacizumab IC_{50} (μg/mL)*
B16-F10	Melanoma	5.5	NR
YUMMER1.7	Melanoma	3.5	NR

*Mean of 4 replicates. NR=Not reached (>100 μM lenvatinib or >300 μg/mL bevacizumab)

Murine melanoma cultures utilized in vivo also demonstrate in vitro superior direct cytotoxic activity of lenvatinib compared to bevacizumab.

Supplemental Table 4: Flow cytometry antibody panel.

Color	Antibody	Company	Catalog	Dilution	Isotype	Company	Catalog	Dilution
BUV395	F4/80	BD	565614	1:100	Rat IgG2a, κ	BD	563556	1:100
BUV395	CD3	BD	740268	1:200	Rat IgG2b, κ	BD	563560	1:200
BUV395	CD19	BD	563557	1:200	Rat IgG2a, κ	BD	563560	1:200
BUV395	TCR	BD	742485	1:200	Armenian Hamster IgG2, λ1	BD	565820	1:200
BUV 737	CD45	BD	612778	1:1000	Mouse IgG2a, κ	BD	612765	1:1000
BV421	CD64	BioLegend	139309	1:100	Mouse IgG1, κ	BioLegend	400157	1:100
BV421	CXCR3	BioLegend	155907	1:100	Rat IgG2b, κ	BioLegend	400639	1:100
BV421	CD62L	BioLegend	104436	1:100	Rat IgG2a, κ	BioLegend	400535	1:100
AmCyan	Live/Dead	Thermo Fisher	L34965	1:1000	Not Applicable			
FITC	CCR2	Thermo Fisher	PA5-23046	1:100	Rabbit / IgG	BioLegend	406404	1:100
FITC	CD44	BioLegend	103006	1:100	Rat IgG2b, κ	BioLegend	400605	1:100
FITC	CD172a	BioLegend	144006	1:100	Rat IgG1, κ	BioLegend	400405	1:100
Percp-cy5.5	Ly6C	BioLegend	128012	1:500	Rat IgG2c, κ	BioLegend	400723	1:500
Percp-cy5.5	CD8	BioLegend	100734	1:250	Rat IgG2a, κ	BioLegend	400531	1:250
Pe-Cy7	CD11c	BioLegend	117318	1:200	Armenian Hamster IgG	BioLegend	400921	1:200
Pe-Cy7	Ki-67	ebiosceince	25-5698-82	1:250	Rat IgG2a, κ	BioLegend	400521	1:250
Pe-Cy7	CCR7	BioLegend	120124	1:100	Rat IgG2a, κ	BioLegend	400521	1:100
Pe-Cy7	CD169	BioLegend	142412	1:100	Rat IgG2a, κ	BioLegend	400521	1:100
PE	iNOS	BioLegend	696806	1:100	Rat IgG2b, κ	BioLegend	400635	1:100
PE	PD1	BioLegend	114118	1:100	Rat IgG2a, κ	BioLegend	400507	1:100
BV605	Ly6G	BioLegend	127639	1:100	Rat IgG2a, κ	BioLegend	400539	1:100
APC	CD206	BioLegend	141708	1:100	Rat IgG2a, κ	BioLegend	400511	1:100
APC	XCR1	BioLegend	148206	1:200	Mouse IgG2b, κ	BioLegend	400319	1:200
APC	Foxp3	Millipore Sigma	MABF730	1:100	Mouse IgG1κ	BioLegend	400119	1:100
APC	CD103	BioLegend	121414	1:100	Armenian Hamster IgG	BioLegend	400911	1:100
APC-Cy7	CD43	BioLegend	121220	1:100	Rat IgG2a, κ	BioLegend	400523	1:100
APC-Cy7	CD11b	BioLegend	101226	1:100	Rat IgG2b, κ	BioLegend	400623	1:100
AF700	MHC II	BioLegend	107622	1:400	Rat IgG2b, κ	BioLegend	400628	1:400
AF700	CD4	BioLegend	100430	1:100	Rat IgG2b, κ	BioLegend	400628	1:100
AF700	CD69	BioLegend	104539	1:100	Armenian Hamster IgG	BioLegend	400926	1:100
